# Atherosclerosis-associated hepatic secretion of VLDL but not PCSK9 is dependent on cargo receptor protein Surf4

**DOI:** 10.1016/j.jlr.2021.100091

**Published:** 2021-06-09

**Authors:** Bingxiang Wang, Yishi Shen, Lei Zhai, Xiaodan Xia, Hong-mei Gu, Maggie Wang, Yongfang Zhao, Xiaole Chang, Adekunle Alabi, Sijie Xing, Shijun Deng, Boyan Liu, Guiqing Wang, Shucun Qin, Da-wei Zhang

**Affiliations:** 1 Institute of Atherosclerosis and College of Basic Medical Sciences in Shandong First Medical University (Shandong Academy of Medical Sciences), Taian, China; 2 Department of Pediatrics and Group on the Molecular and Cell Biology of Lipids, Faculty of Medicine and Dentistry, University of Alberta, Edmonton, Alberta, Canada; 3 Department of Orthopedics, The Sixth Affiliated Hospital of Guangzhou Medical University, Qingyuan People's Hospital, Qingyuan, China

**Keywords:** apolipoproteins, LDL, lipid metabolism, lipoprotein metabolism, liver, triglyceride, stearyl-CoA-1, apoB100, LDLR, AAV, adeno-associated virus, Alb, albumin, ALT, alanine aminotransferase, apoB, apolipoprotein B, apoB100, apolipoprotein B100, ASCVD, atherosclerotic cardiovascular disease, CE, cholesteryl ester, COPII, classical coat protein complex II, LDLR, LDL receptor, MTP, microsomal triglyceride transfer protein, PCSK9, proprotein convertase subtilisin/kexin type 9, SCD1, stearoyl-CoA desaturase-1, Surf4, Surfeit 4, *Surf4*^LKO^, Surf4 liver-specific knockout, TANGO1, Transport and Golgi Organization 1, TC, total cholesterol, TG, triglyceride, VTV, VLDL transport vesicle

## Abstract

Plasma LDL is produced from catabolism of VLDL and cleared from circulation mainly via the hepatic LDL receptor (LDLR). Proprotein convertase subtilisin/kexin type 9 (PCSK9) promotes LDLR degradation, increasing plasma LDL-C levels. Circulating PCSK9 is mainly secreted by the liver, whereas VLDL is exclusively secreted by hepatocytes. However, the mechanism regulating their secretion is not completely understood. Surfeit 4 (Surf4) is a cargo receptor localized in the ER membrane. It recruits cargos into coat protein complex II vesicles to facilitate their secretion. Here, we investigated the role of Surf4 in VLDL and PCSK9 secretion. We generated Surf4 liver-specific knockout mice and found that knockout of Surf4 did not affect PCSK9 secretion, whereas it significantly reduced plasma levels of cholesterol, triglyceride, and lipid-binding protein apolipoprotein B (apoB). In cultured human hepatocytes, Surf4 coimmunoprecipitated and colocalized with apolipoprotein B100, and Surf4 silencing reduced secretion of apolipoprotein B100. Furthermore, knockdown of Surf4 in LDLR knockout (*Ldlr*^−/−^) mice significantly reduced triglyceride secretion, plasma levels of apoB and non-HDL-C, and the development of atherosclerosis. However, Surf4 liver-specific knockout mice and Surf4 knockdown in *Ldlr*^−/−^ mice displayed similar levels of liver lipids and plasma alanine aminotransferase activity as control mice, indicating that inhibition of Surf4 does not cause notable liver damage. Expression of stearoyl-CoA desaturase-1 was also reduced in the liver of these mice, suggesting a reduction in de novo lipogenesis. In summary, hepatic deficiency of Surf4 reduced VLDL secretion and the development of atherosclerosis but did not cause significant hepatic lipid accumulation or liver damage.

VLDL is a triglyceride (TG)-rich lipoprotein exclusively generated and secreted from the liver. TG in VLDL is hydrolyzed by LPL, leading to the formation of IDL that can be further metabolized to LDL (1, 2). Plasma LDL-C levels are positively correlated with the risk of atherosclerotic cardiovascular disease (ASCVD), one of the leading causes of morbidity and mortality worldwide. Also, emerging evidence indicates that elevated levels of remnant cholesterol, such as VLDL and IDL, are associated with an increased risk of ASCVD (3). It has also been reported that dysregulation of VLDL secretion occurs under many pathophysiological conditions. For example, insulin resistance and obesity can cause VLDL overproduction, increasing the risk for ASCVD (4). Furthermore, patients with homozygous familial hypercholesterolemia harboring LDL receptor (LDLR) null mutations or with autosomal recessive hypercholesterolemia cannot be effectively treated by current available lipid-lowering drugs, such as statins and proprotein convertase subtilisin/kexin type 9 (PCSK9) inhibitors (5). Inhibition of VLDL secretion can markedly reduce plasma levels of cholesterol and the development of atherosclerosis (6, 7, 8, 9, 10). However, the biggest challenge is that current strategies to inhibit VLDL secretion usually lead to hepatic TG accumulation and subsequent fatty liver and liver steatosis as VLDL secretion is the primary route of TG export from the liver (6, 7, 8, 11). Thus, deciphering the molecular mechanism of VLDL secretion not only advances our understanding of the regulation of lipid metabolism but also is essential for the identification of novel therapeutic targets with minimal side effects.

Newly synthesized apolipoprotein B100 (apoB100) is cotranslationally lipidated in the lumen of the ER of hepatocytes by microsomal triglyceride transfer protein (MTP). Unlipidated apoB100 is rapidly degraded via the proteasome-dependent and proteasome-independent pathways (1, 12). After assembly in the ER lumen, VLDL is transported to the Golgi apparatus through specific VLDL transport vesicles (VTVs) as VLDL is too large to utilize the classical coat protein complex II (COPII) vesicles (1, 13). Recently, Santos *et al.* (13) reported that Transport and Golgi Organization 1 (TANGO1) and TANGO1-Like protein were required for the formation of VTV and VLDL secretion in HepG2 cells. TANGO1 was also required for the export of other bulky molecules, such as collagens, via bringing them to the ER exit site (13, 14, 15, 16). Both TANGO1 and TANGO1-Like protein contain an N-terminal luminal SH3-like domain that is required for secretion of bulky cargos (13). However, how does the SH3-like domain recognize various kinds of bulky cargos such as collagens and VLDL? It has been reported that the SH3-like domain of TANGO1 does not directly interact with collagens. Instead, heat shock protein 47 binds to collagens and the SH3-like domain in TANGO1, bridging the interaction between collagens and TANGO1 (16). It is currently unknown whether such a protein is required for sorting VLDL into VTV. However, understanding this process can provide critical information for the development of novel LDL production-based therapies.

Surfeit 4 (Surf4) is ubiquitously expressed and localized in the ER membrane. It consists of five putative transmembrane domains, a C-terminal cytosolic domain that associates with SEC24 in COPII, and an ER luminal region that recognizes and binds to luminal cargos. Surf4 acts as a cargo receptor to recruit substrates into COPII vesicles, facilitating their secretion (17, 18, 19). However, the physiological substrates of Surf4 are still not well understood. Recently, Emmer *et al.* (20) observed that Surf4 facilitated the secretion of PCSK9 overexpressed in human embryonic kidney 293 cells. In contrast, we found that Surf4 was not required for endogenous PCSK9 secretion from cultured human hepatoma-derived cell lines, Huh7 and HepG2 cells (21). PCSK9 promotes LDLR degradation and plays a central role in regulating the plasma level of LDL (22, 23, 24). To further determine the role of Surf4 in PCSK9 secretion, we generated Surf4 liver-specific knockout (*Surf4*^LKO^) mice and found that a lack of hepatic Surf4 had no significant effect on PCSK9 secretion but significantly reduced VLDL secretion. Furthermore, knockdown of Surf4 drastically reduced the development of atherosclerosis in *Ldlr*^−/−^ mice but did not cause hepatic lipid accumulation or notable liver damage.

## Materials and methods

### Materials

DMEM, FBS, BSA, and cOmplete™ EDTA-free protease inhibitors were purchased from Millipore Sigma or Hyclone. Penicillin-streptomycin, trypsin-EDTA solution, Lipofectamine® 3000, Lipofectamine® RNAiMAX, High Capacity cDNA Reverse Transcription Kit, SYBR® Select Master Mix, PureLink™ Hipure Plasmid Miniprep and Maxiprep Kits, BCA Protein Assay Kit, TRIzol®, Alexa Fluor 488 donkey anti-rabbit IgG, Alexa Fluor 647 goat anti-mouse IgG, and Alexa Fluor 647 donkey anti-goat IgG were obtained from Thermo Fisher Scientific. Quick ligation kit, T4 DNA polymerase, and high-fidelity restriction enzymes were purchased from New England Biolabs. Mouse PCSK9 ELISA Kit was from abcam. RNeasy® Mini Kit was from Qiagen. All other reagents were obtained from Fisher Scientific unless otherwise indicated.

Scrambled and predesigned Dicer-Substrate siRNA were purchased from IDT® and listed in Table 1. The antibodies are listed in Table 2. The custom-made rabbit polyclonal anti-Surf4 antibody, 1195Sa, was produced and purified by GenScript® using a peptide that is completely conserved among different species (21).

**Table 1:** List of primers

1. Genotyping i. 5′ Loxp: Forward (F): 5′-AATGCTGCTTGTGGCATCTCAAAGG-3′; Reverse (R): 5′-CTACCAGGT TTGTTCCACCCTCCAA-3′ ii. 3′ Loxp: F: 5′-CCAGAAAGGACAAAAGGGTTCAGTC-3′; 5′-TACAAGGCCTTT CTCACCTCCTAA C-3′ iii. Alb-Cre gene: 20239: 5′-TGCAAACATCACATGCACAC-3′; 20240: 5′-TTGGCCCCTTACCATAACTG-3′; 5′-GAAGCAGAAGCTTAGGAAGATGG-3′ 2. DsiRNA Scrambled DsiRNA: F: 5′-AUUAGUGUGCGAUGUACCCAGGAAC-3′; R: 5′-GUUCCUGGGUACAUCGCACACUAAUAU-3′ Human Surf4 DsiRNA1: F: 5′-CGCAUUGGUAUUAUCAUUCAAAGCA-3′; R: 5′-UGCUUUGAAUGAUAAUACCAAUGCGUC-3′ Human Surf4 DsiRNA2: F: 5′-AUGACUUCCUGAAAUACGACUUCTT-3′; R: 5′-AAGAAGUCGUAUUUCAGGAAGUCAUGC-3′ 3. Quantitative real-time PCR primers *Gapdh* F: 5′-AACTTTGGCATTGTGGAAGG-3′; R: 5′-GGATGCAGGGATGATGTTCT-3′ *ApoE* F: 5′-GGCAAACCTGATGGAGAAGATA-3′; R: 5′-TTGTTGCAGGACGGAGAAG-3′ *ApoB* F: 5′-AGGCTTGTCACCCTTCTTTC-3′; R: 5′-GCCTTGTGAGCACCAGTATTA-3′ *Srebf1c* F: 5′-ATCGGCGCGGAAGCTGTCGGGGTAGCGTC-3′; R: 5′-ACTGTCTTGGTTGTTGATGAGCTGGAGCAT-3′ *Scd1* F: 5′-CTGACCTGAAAGCCGAGAAG-3′; R: 5′-AGAAGGTGCTAACGAACAGG-3′ *Fasn* F: 5′-CCCCTCTGTTAATTGGCTCC-3′; R: 5′-TTGTGGAAGTGCAGGTTAGG-3′ *Agpat1* F: 5′-CAGCTCCAGTGCCAAGTATT-3′; R: 5′-GGAACTCTGGTGGTTGGAC-3′ *Ggat1* F: 5′-GGATCTGAGGTGCCATCGT-3′; R: 5′-CCACCAGGATGCCATACTTG-3′ *Ggat2* F: 5′-GGCTACGTTGGCTGGTAACTT-3′; R: 5′-TTCAGGGTGACTGCGTTCTT-3′ *Gpat1* F: 5′-CAACACCATCCCCGACATC-3′; R: 5′-TTTTTCCGCAGCATTCTGATAA-3′ *Srebf2* F: 5′-CCCTATTCCATTGACTCTGAGC-3′; R: 5′-CACATAAGAGGATTCGAGAGCG-3′ *Hmgcr* F: 5′-GCCCTCAGTTCAAATTCACAG; R: 5′-TTCCACAAGAGCGTCAAGAG-3′ *Ldlr* F: 5′-ACCCGCCAAGATCAAGAAAG; R: 5′-GCTGGAGATAGAGTGGAGTTTG-3′ *Surf4* F: 5′-CAGCGTGACTATATCGACACC-3′; R: 5′-GATTCCAAACAGCCCAAAGC-3′ *Ppar-α* F: 5′-CATTTCCCTGTTTGTGGCTG-3′; R: 5′-ATCTGGATGGTTGCTCTGC-3′ *Acad1* F: 5′-GGTGGAAAACGGAATGAAAGG-3′; R: 5′-GGCAATCGGACATCTTCAAAG-3′ *Cpt1a* F: 5′-AGACAAGAACCCCAACATCC-3′; R: 5′-CAAAGGTGTCAAATGGGAAGG-3′ 4. shRNA i. Scrambled shRNA: 5′-GTATGCAGCGGTATCGTGTTG-3′ ii. Surf4 shRNA: 5′-GGGACTTGAAGTTTCTCATGA-3′

**Table 2:** The list of antibodies

Name	Company	Host	Catalog no.	Dilution	Notes
ABCA1	abcam	Mouse	ab18180	1:1,000	WB
Β-Actin	Bioss	Rabbit	Bs-0061R	1:2,000	WB
Β-Actin	BD Biosciences	Mouse	612657	1:5,000	WB
Albumin	abcam	Rabbit	ab207327	1:1,000	WB
ApoAI	abcam	Rabbit	ab227455	1:1,000	WB
ApoAI	Cell Signaling	Mouse	3350	1:1,000	WB
ApoAI	Bioss	Rabbit	Bs-0849R	1:1,000	WB
ApoB	abcam	Rabbit	ab20737	1:1,000	WB
ApoB	MilliporeSigma	Goat	AB742	1:2,000	WB
ApoB	abcam	Goat	ab7616	1:100	IF/IP
ApoE	abcam	Mouse	ab1906	1:1,000	WB
Calnexin	abcam	Rabbit	ab22595	1:1,000	WB
CPT1A	abcam	Mouse	ab128568	1:1,000	WB
GRP78	abcam	Rabbit	ab108615	1:1,000	WB
HMGCR	abcam	Rabbit	Ab174830	1:1,000	WB
LDLR	abcam	Rabbit	ab52818	1:1,000	WB
MTP	Bioworld	Rabbit	BS-6672	1:1,000	WB
PCSK9	abcam	Rabbit	ab31762	1:1,000	WB
SCD1	abcam	Rabbit	ab236868	1:1,000	WB
Surf4	Custom-made (GenScript)	Rabbit	N/A	1:2,000/1:100/1:200	WB/IP/IF (21)
Transferrin receptor	BD Biosciences	Mouse	612125	1:1,000	WB

IF, immunofluorescence; IP, immunoprecipitation; N/A, not available; WB, Western blot.

### Animal

The WT C57Bl/6J, *Ldlr*^−/−^, and *Pcsk9*^−/−^ mice were purchased from the Jackson Laboratory and then housed and bred in the animal facility at the University of Alberta. *Surf4*^Flox^ mice in C57BL/6 background were generated in Biocytogen (Beijing, China). *Sur4*^Flox^ and *Surf4*^LKO^ mice were maintained in the animal facility at Shandong First Medical University (Taian, China). Mice used in all experiments were 10–14 weeks old and fasted for 10 h unless otherwise indicated.

Three to five mice were housed per cage with free access to water in a climate-controlled facility with a 12-h light/dark cycle. After weaning, mice were fed ad libitum a chow diet containing 20% protein, 5% fat, and 48.7% carbohydrates (Keao Xieli, Beijing, China, or LabDiet, PICO Laboratory Rodent Diet 20). For the high fat/high cholesterol experiment, mice were fed the Western-type diet containing 0.15% cholesterol (TestDiet 1813029; kilocalorie from fat 40%, protein 16%, and carbohydrate 44%). All animal procedures were approved by the University of Alberta's Animal Care and Use Committee and conducted in accordance with guidelines of the Canadian Council on Animal Care or approved by Shandong First Medical University's Animal Care and Use Committee.

### Cell culture, transfection, and immunoblotting

Primary hepatocytes were isolated as described (25). The liver was first perfused with HBSS buffer containing 0.5 mM EGTA and then HBSS containing 1 mg/ml of collagenase (Sigma, Burlington, MA) for 6–10 min before collection. The isolated hepatocytes were seeded on collagen-coated dishes in DMEM (high glucose) containing 15% FBS for 4 h. Afterward, nonadherent cells were removed, and the hepatocytes were incubated in fresh DMEM (high glucose) containing 15% FBS for up to 48 h.

Huh7 and HepG2 cells were maintained in DMEM (high glucose) containing 10% (v/v) FBS at 37°C in a 5% CO_2_ humidified incubator. Dicer-Substrate siRNA was introduced into cells using Lipofectamine™ RNAiMAX according to the manufacturer's instruction. About 72 h after transfection, the cells were washed twice in PBS, collected, and then lysed in lysis buffer A (1% Triton, 150 mM NaCl, 50 mM Hepes, and pH 7.4) with 1× cOmplete mini EDTA-free protease inhibitors for 30 min on ice. After centrifugation for 15 min at 20,000 *g* at 4°C, the supernatant was collected as whole cell lysate.

Liver samples were collected from euthanized mice, homogenized in RIPA buffer (50 mM Tris, pH 7.4, 150 mM NaCl, 1% Triton X-100%, 1% sodium deoxycholate, 0.1% SDS, sodium orthovanadate, sodium fluoride, EDTA, leupeptin, and PMSF), and centrifuged at 20,000 *g* at 4°C for 20 min. The supernatant was harvested as tissue homogenate. Protein concentrations of whole cell lysate and tissue homogenate were determined by the BCA protein assay. Equivalent amounts of lysate proteins were applied to SDS-PAGE and then transferred to nitrocellulose (GE Healthcare) or PVDF membranes (Millipore) by electroblotting. Immunoblotting was performed using specific antibodies as indicated. Antibody binding was detected using IRDye® 680 or IRDye® 800-labeled donkey anti-mouse, anti-rabbit, or anti-goat IgG (Li-Cor), followed by imaging on a Licor Odyssey Infrared Imaging System (Li-Cor). Alternatively, antibody binding was detected by HRP-conjugated goat anti-mouse or anti-rabbit IgG antibody (abcam), followed with Pierce™ ECL Western Blotting Substrate and X-ray film exposure.

### Immunoprecipitation analysis

Immunoprecipitation was performed as described before (21, 26). When the confluency reached about 80%, cells in two 100 mm dishes were lysed in 1,000 μl of lysis buffer A containing 1× cOmplete EDTA-free protease inhibitors. About 50 μl of cell lysate was saved for Western blot. Equal amounts of total protein were applied to a rabbit anti-Surf4 or preimmune serum and protein G beads. Immunoprecipitated samples were washed three times with lysis buffer A. The immunoprecipitated proteins were then eluted from the beads by addition of 2× SDS-PAGE sample buffer (100 mM Tris-HCl, pH 6.8%, 4% SDS, 20% glycerol, and 0.04% bromophenol) containing 5% β-mercaptoethanol. Equal amounts of eluted samples and whole cell lysate were applied to SDS-PAGE and immunoblotting.

### Immunofluorescence

Confocal microscopy was carried out as described (27, 28, 29). Huh7 cells were seeded onto coverslips (1.0 × 10^5^ cells/ml). About 48 h later, cells were fixed and permeabilized with cold methanol for 10 min at −20°C. The cells were then incubated with a goat anti-apoB polyclonal antibody and rabbit anti-Surf4 antibody overnight at 4°C. Antibody binding was detected using Alexa Fluor 647 donkey anti-rabbit IgG and Alexa Fluor 488 donkey anti-goat IgG. Nuclei were stained with 4′,6-diamidino-2-phenylindole (Thermo Fisher). After washing, coverslips were mounted on the slides with ProLong Diamond Antifade Mountant (Thermo Fisher). Localizations of apoB and Surf4 were determined using a Leica SP5 laser scanning confocal microscope (filters: 461 nm for 4′,6-diamidino-2-phenylindole, 519 nm for Fluor 488, and 633 nm for Fluor 647).

### Quantitative real-time PCR

Total RNAs were extracted from mouse tissues using TRIzol® according to the manufacturer's protocols. cDNA was synthesized using a RT kit CW2019M from Cwbiotech Company (China). Relative quantitative real-time PCR was carried out on StepOnePlus™ using SYBR® Select Master Mix according to the manufacturer's instruction. Relative gene expression was normalized to *Gapdh*. Primers were designed by PrimerQuest Real-Time PCR Design Tool, synthesized by IDT, Inc. and listed in Table 1.

### Histochemistry

Fresh liver tissues were embedded in optimal cutting temperature compound, cut into 8 μm thick cryostat sections, and then mounted on slides. The sections were fixed in 10% neutral buffered formalin for 10 min. The fixed sections were soaked in 60% isopropanol for 20 s, stained with Oil Red O for 15 min, rinsed twice in 60% isopropanol, and then soaked in hematoxylin to stain nuclei. Afterward, the sections were sealed with gelatin. All slices were imaged on a microscope (Nikon, Tokyo, Japan). Relative stained areas were quantified with ImageJ software (National Institutes of Health) using color segmentation and threshold analysis.

For H&E staining, liver tissues were fixed in neutral buffered formalin and dehydrated through a series of increasing concentrations of ethanol (70, 95, and 100%) at room temperature. The samples were then embedded in paraffin, cut into 8 μm thick sections, and mounted on slides. The sections were subsequently deparaffinized, rehydrated, and then washed in 1× PBS three times. The slides were then soaked in the hematoxylin for 3–5 s and differentiated in 1% ethanol hydrochloride for 1–2 s, followed by an immediate wash with running water for 10–20 s. After soaking in 0.5% eosin stain for 5 s and washing with water, the slides were dehydrated by immersion in ethanol solutions of different concentrations sequentially, cleared in xylene, and then sealed with neutral gum for microscopic observation. The histological scores were evaluated blindly.

### Plasma lipid, ALT, and PCSK9 analysis

Blood samples were collected into EDTA-coated tubes from mice fasted overnight (10 h) and then centrifuged at 3,000 *g* for 10 min. Plasma from each mouse was subjected to analysis of alanine aminotransferase (ALT), TG, total cholesterol (TC), HDL-C, and non-HDL-C, using their specific kits according to the manufacturer's instructions (Applygen, China; FIJIFILM Wako Diagnostics; Cayman Chemical Company).

Plasma PCSK9 (1:4 diluted in the reagent diluent) was assessed using the mouse PCSK9 ELISA Kit in accordance with manufacturer's protocol (abcam). About 50 μl of standards or diluted samples and 100 μl of HRP-labeled detection antibody were added to an assay plate coated with the capture antibody and then blocked in reagent diluent. Following a 1 h incubation period at 37°C, the plate was washed with reagent detergent five times. Substrates A and B were then added to each well and incubated for 15 min at 37°C. The reaction was terminated by adding 50 μl of stop solution. The absorbance was measured using a SpectraMax i3x plate reader at a wavelength of 450 nm.

### ApoB and TG secretion and LDL/VLDL clearance

The cells were washed twice with DMEM without FBS and then incubated in DMEM containing 0.4 mM oleic acid complexed to 0.5% FA-free BSA (Sigma) for 4 h. The cells were then washed twice in DMEM without FBS and incubated in DMEM without oleic acid and FBS for 16 h. Culture medium and cells were collected separately, and whole cell lysate was prepared. Equal amounts of total proteins in culture medium or whole cell lysate were applied to immunoblotting.

TG secretion was measured as described (30). Mice were fasted for 10 h and then injected with poloxamer-407 dissolved in saline (100 mg/kg body weight) intraperitoneally. Approximately 100 μl of blood sample was collected before and 0.5, 1, 2, and 4 h postinjection. Plasma TG was measured using a commercial colorimetric kit (Applygen, China).

The clearance rate of LDL and VLDL was determined as previously described (31). LDL and VLDL were labeled with ^125^I (PerkinElmer) using precoated iodination tubes (Thermo Fisher) according to the manufacturer's instructions. Briefly, 1 mg of LDL or VLDL protein and 0.5 mCi Na^125^I in PBS were added to the precoated iodination tubes. The reaction mixture (200 μl) was gently shaken at 1 min intervals at room temperature for 10 min. Unlabeled ^l25^I was removed using a desalting column (catalog no. 43243; Thermo Fisher). Fractions containing ^l25^I-LDL or ^l25^I-VLDL were pooled together. Radioactivity and protein concentrations were measured by γ counting and BCA, respectively. Mice were administered with ^l25^I-LDL or ^l25^I-VLDL (∼15 μg per mouse). Blood was collected at 2 min, 30 min, 1 h, 2 h, and 4 h after injection. Plasma samples were combined with equal volumes of 100% isopropanol, vortexed, incubated overnight at room temperature, and then centrifuged at 1,000 *g* for 30 min. The pellets were washed twice with 50% isopropanol and then dissolved in 15–30 μl of NaOH. Radioactivity of the samples was measured immediately in a γ spectrometer.

### FA oxidation

The experiments were carried out exactly according to the manufacturer's protocol (abcam; ab222944). Briefly, primary hepatocytes were isolated from *Surf4*^Flox^ and *Surf4*^LKO^ mice. Cells were then seeded in a 96-well plate at the density of 6 × 10^4^ cells/well and incubated overnight. The cells were washed twice with prewarmed FA-free measurement media, incubated with FA measurement media and extracellular O_2_ consumption reagent, and then sealed with prewarmed high-sensitivity mineral oil. The fluorescence signal was read in a microplate reader (Ex/Em = 380/650 nM).

### Measurement of liver lipids

Lipid content in the liver was measured using colorimetric kits from Applygen (Beijing, China) according to the manufacturer's instructions. About 1 μg of liver tissue was homogenized with 900 μl of anhydrous ethanol on ice and subsequently centrifuged at 660 *g* for 10 min at 4°C. The supernatant was collected. Protein concentration was determined via the BCA assay. TG and TC levels were determined using their specific commercial enzymatic kits. The absorbance was measured using a SpectraMax i3x Microplate Reader.

Lipids were also extracted from the liver using the Folch method (32). Liver samples were homogenized in 50 mM Tris-HCl (pH 7.4), 250 mM sucrose, and 1 mM EDTA by a glass/Teflon homogenizer for 20 s. Lipids were extracted from 4 mg of liver homogenate with chloroform:methanol (2:1). Samples were sonicated 3 × 10 s on ice, and the volume was topped to 1 ml with PBS. Homogenized samples were then transferred into a glass tube with 4 ml of chloroform/methanol (2:1) and incubated for 1 h with vigorous agitation. The extraction was spun at 1,000 *g* for 10 min, and the bottom phase was transferred to a new glass tube and dried under nitrogen. Lipids were dissolved in 1 ml of chloroform with 2% Triton X-100. Afterward, the samples were dried under nitrogen and dissolved in 1 ml of distilled water. TG and TC from 50 μl aliquots of the sample were measured using commercial kits from FUJIFILM WAKO and Cell Biolabs.

Liver lipids were further measured using LC-MS/MS. Lipids were extracted from the liver using the methyl-tert-butyl ether method with modifications. Liver tissues (8–10 mg) were homogenized in 280 μl of cold methanol containing an internal standard mixture (cholesteryl ester [CE 17:0, triacylglycerol TG 17:0/17:1/17:0, and *d7*-cholesterol). About 50 μl of homogenate was used to measure protein concentrations with the BCA Protein Assay (Solarbio Co., Beijing, China). About 1.0 ml of methyl-tert-butyl ether was added to the remaining homogenate. The samples were then vortexed and transferred to a rotary spinner at 40 rpm for 1 h at room temperature. Phase separation was induced by adding 325 μl of distilled water. After vortex, samples were centrifuged at 10,000 *g* for 10 min at 4°C. About 350 μl of the upper hydrophobic fraction was transferred to a new tube, dried under nitrogen, and stored at −80°C. The lipid extraction was reconstituted in 200 μl of acetonitrile:2-propanol (1:1, v/v) prior to LC-MS/MS analysis.

LC-MS/MS was performed using a Shimadzu LC-20 AD binary pump system coupled to a SIL-20AC autoinjector and interfaced with an ABI 4000 QTrap mass spectrometer (Sciex, Framingham, MA). Chromatographic separation was carried out on a Waters Symmetry C18 column (3.5 μm, 2.1 mm i.d. × 100 mm) with a Waters C18 guard column (3.5 μm, 2.1 mm i.d. × 10 mm) at 40°C. The injection volume was 10 μl with a flow rate of 0.3 ml/min. The mobile phase consisted of (A) 10 mM ammonium formate in acetonitrile:water:formic acid (83:17:0.1, v/v/v) and (B) 10 mM ammonium formate in acetonitrile:2-propanol:formic acid (50:50:0.1, v/v/v). Isocratic elution was performed with 95% B for 16 min. Detection was accomplished by using a multiple reaction monitoring mode with positive ion detection. For CE and TG, electrospray ionization source was selected with the following settings: ion spray voltage = 5,500 V, ion source heater temperature = 400°C, source gas 1 = 40 psi, source gas 2 = 40 psi, and curtain gas = 10 psi. For free cholesterol, atmospheric pressure chemical ionization source was selected with a nebulizer gas pressure of 55 psi and a curtain gas pressure of 20 psi, 3 μA nebulizer current, 550°C source temperature, and medium nitrogen collision gas pressure. Relative quantification of lipids was based on the intensity of each lipid divided by the intensity of the internal standard and then normalized to protein concentrations.

### Electron microscopy and NMR

Ultrathin sections of liver samples were prepared and scanned by Wuhan Servicebio Technology Company (Wuhan, China). The livers were fixed in 4% paraformaldehyde for 2 h and washed in PBS and double-distilled water three times for 30 min. The samples were then dehydrated for 1 h in increasing concentrations of ethanol (30, 50, 70, 90, and 100%) at room temperature. Afterward, the samples were immersed in resin K4M (ethanol:K4M = 2:1 for 12 h, ethanol:K4M = 1:1 for 12 h, and K4M for 12 h) and then transferred to SBI-beam capsules. The resin was polymerized under UV light for 12 h at room temperature, 24 h at 0°C, and 24 h at −30°C. Sections were cut on a Reichert-Jung-B ultracut, collected on Formvarc-covered copper grids, and observed under a HT7700 transmission electron microscope.

Mice (12 weeks old) were scanned noninvasively on a minispec NMR analyzer (LF50; Bruker). Data were collected, analyzed by the analyzer, and then presented as the ratio of fat or lean mass to body weight.

### Adeno-associated virus preparation

Adeno-associated virus (AAV) containing scrambled shRNA or shRNA against mouse Surf4 was prepared using the AAV-DJ/8 Helper Free Expression System according to the manufacturer's instruction (Cell Biolabs, Inc.; VPK-410-DJ-8). shRNAs against mouse *Surf4* and scrambled shRNA were designed by BLOCK-iT™ RNAi Designer and synthesized by IDT with a 9-nucleotide hairpin loop sequence (5′-TTCAAGAGA-3′) in the middle, a 5′-BamHI restriction site overhang on the top strand, and a 5′-EcoRI restriction site overhang on the bottom strand (Table 1). shRNA was then cloned into the BamHI/EcoRI sites of pAAV-U6-GFP. AAV was packaged and amplified in QBI 293A cells and then purified using Optiprep (Sigma) density-gradient ultracentrifugation as described (33). AAV particles were collected from the 40% density step, diluted in PBS, concentrated with Amicon Ultra-15 Centrifugal Filter Unit (Millipore; 100K NMWL), and titered using quantitative real-time PCR.

### Atherosclerotic analysis

Male *Ldlr*^−/−^ mice (8–12 weeks of age) were randomly divided into two groups (six mice per group) and injected with AAV-U6-Scrambled shRNA (control group) or AAV-U6-*Surf4* shRNA via retroorbital injection (2.0 × 10^10^ genomic copy/mouse). The mice were then fed the Western-type diet containing 0.15% cholesterol (TestDiet) for 14 weeks. After euthanasia, aortas and hearts were collected immediately and fixed in 4% paraformaldehyde. Serial sections (10 μm thick) throughout the three aortic valves of each mouse heart and aortas were used for Oil Red O staining and analysis. Images were taken using an OMAX M837ZL-C140U3 microscope. The atherosclerotic burden was quantified by measuring the surface area of Oil Red O-positive lesions on the cross-sectional area of the aorta sinus. Lesion areas were quantified with OMAX ToupView.

### Statistical analysis

All statistical analyses were carried out by GraphPad Prism, version 9.0 (GraphPad Software). The significant differences between groups were determined via Student's *t*-test. All data met normal distribution criteria, and variance between groups that was analyzed by F-test showed no significant difference (*P* > 0.05). Values of all data, unless otherwise indicated, were depicted as mean ± SD. The significance was defined as ∗*P* < 0.05, ∗∗*P* < 0.01, ∗∗∗*P* < 0.001, and ∗∗∗∗*P* < 0.0001. All experiments, unless indicated, were repeated at least three times.

## Results

### Generation of *Surf4*^LKO^ mice and PCSK9 secretion

**Fig. 1:**
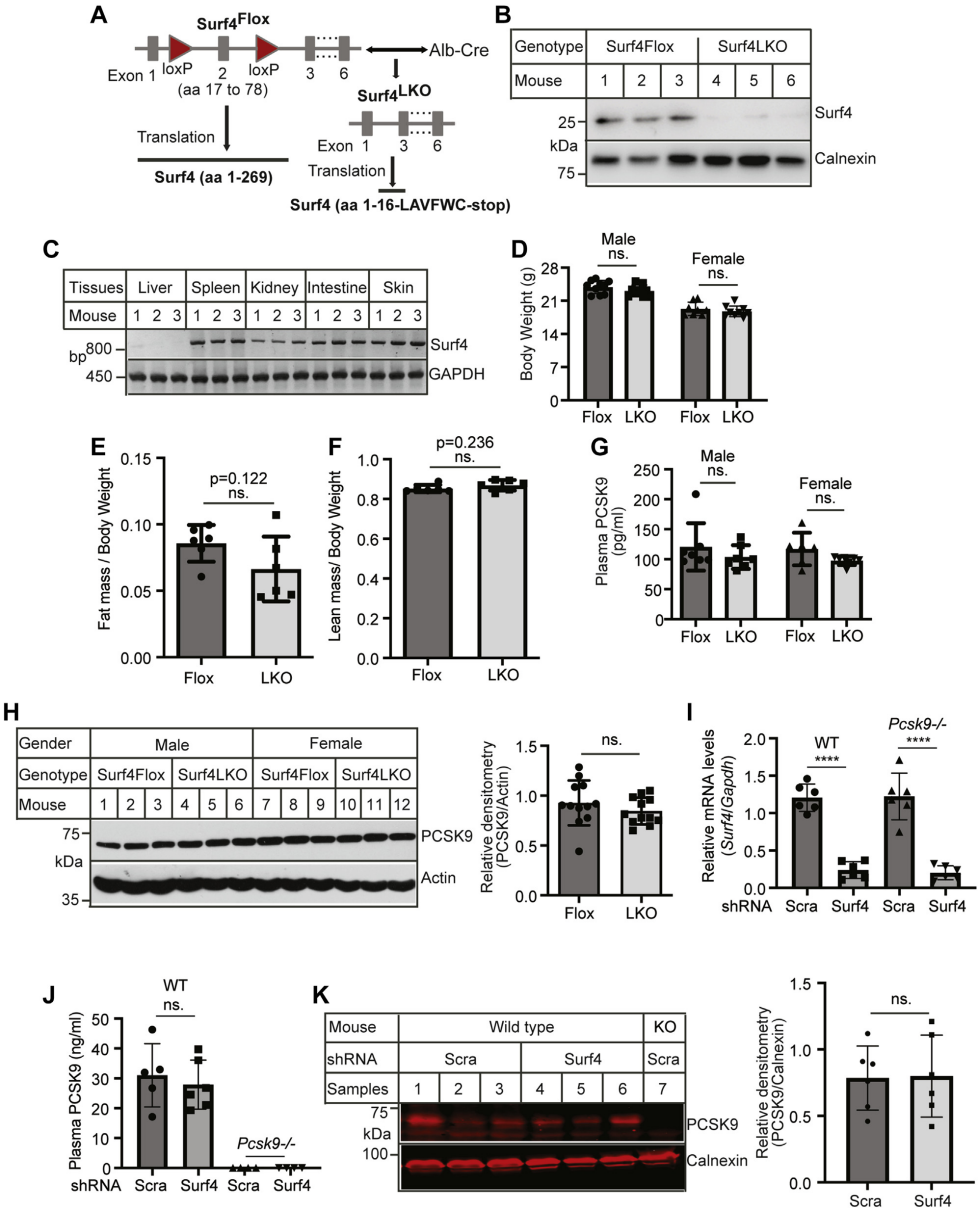
The effect of hepatic knockout of Surf4 on PCSK9 secretion. A: Schematic of *Surf4*^LKO^ mice generation. *Surf4*^Flox^ mice were mated with Alb-Cre mice to generate heterozygous *Surf4*^LKO^ mice, which were then bred to produce homozygous *Surf4*^LKO^ mice. B: Immunoblotting. Whole cell lysate of primary hepatocytes isolated from *Surf4*^Flox^ and *Surf4*^LKO^ mice (*n* = 3) was applied to immunoblotting with antibodies indicated. C: Genotyping. DNA from different tissues of *Surf4*^LKO^ mice (*n* = 3) was used to detect the LoxP-flanked exon 2 of *Surf4*. One of the primers was located within exon 2 between the two LoxP sites. The PCR product was amplified only in the WT or floxed *Surf4* gene but not in *Surf4* gene knockout of exon 2. D–F: Body weight and fat and lean mass. About 14-week-old male and female *Surf4*^*FLOX*^*and Surf4*^*LKO*^ mice on a regular chow diet were fasted for 10 h and then weighed (D, 10 mice per group). Fat (E) and lean mass (F) were measured using NMR (six mice per group). G: Fasting plasma PCSK9 in 14-week-old mice (seven mice per group) was measured using an ELISA kit (abcam). H: Liver PCSK9. Liver homogenate was applied to immunoblotting with antibodies indicated. The relative densitometry was the ratio of the densitometry of PCSK9 to actin at the same condition (*n* = 12). I–K: Knockdown of Surf4. The WT and *Pcsk9*^−/−^ (KO) mice (*n* = 6) were injected with AAV-scrambled (Scra) or Surf4 shRNA and then fed the Western-type diet for 4 weeks. The relative mRNA levels were the ratio of Surf4 mRNA levels to that of *Gapdh* (I). Plasma PCSK9 was measured with an ELSA Kit (R&D Systems) (J). Liver homogenate was applied to immunoblotting to detect PCSK9. The relative densitometry was the ratio of the densitometry of PCSK9 to that of calnexin (K). Student's *t*-test was used to determine the significant differences between groups. Values of all data were mean ± SD. The significance was defined as ∗*P* < 0.05, ∗∗*P* < 0.01, ∗∗∗*P* < 0.001, and ∗∗∗∗*P* < 0.0001. *P* > 0.05. ns, no significant difference.

To study the role of Surf4 in PCSK9 secretion, we generated *Surf4*^LKO^ mice as PCSK9 is primarily secreted from hepatocytes (34). Exon 2 of the *Surf4* gene was flanked by the loxP sites via clustered regularly interspaced short palindromic repeats (CRISPR)-Cas9 (Fig. 1A). We then crossed *Surf4*^Flox^ mice with mice expressing Cre recombinase under the control of the hepatocyte-specific albumin (Alb) promotor to delete functional Surf4 specifically in hepatocytes. The Cre-mediated recombination removed the entire exon 2, which deleted amino acid residues from Phe^17^ to Leu^78^ and caused a frameshift at Thr^79^. This shift introduced six amino acid residues (LAVFWC) after Gln^16^, followed by a stop codon (Fig. 1A). Surf4 protein was essentially undetectable in hepatocytes of *Surf4*^LKO^ mice (Fig. 1B). Genotyping also showed that the *Surf4* gene was undetectable in the liver of *Surf4*^LKO^ mice but was detected in the other tissues of *Surf4*^LKO^ mice (Fig. 1C). *Surf4*^LKO^ and *Surf4*^Flox^ mice displayed similar body weight and fat and lean mass (Fig. 1D–F). Knockout of hepatic Surf4 had no significant effect on plasma PCSK9 levels (Fig. 1G). PCSK9 in liver homogenate was also comparable in the two genotypes (Fig. 1H). To confirm this finding, we knocked down *Surf4* expression in the WT and *Pcsk9*^−/−^ mice using AAV-shRNA. AAV-Surf4 shRNA significantly reduced mRNA levels of *Surf4* in the liver of both the WT and *Pcsk9*^−/−^ mice (Fig. 1I). Silencing of Surf4 had no significant effect on the levels of plasma PCSK9 (Fig. 1J) and liver PCSK9 (Fig. 1K) in the WT mice, while PCSK9 was virtually undetectable in *Pcsk9*^−/−^ mice. Thus, unlike the results found in cultured cells (20, 21), the loss of hepatic Surf4 did not affect the levels of PCSK9 in mouse liver and plasma, suggesting that Surf4 is not required for hepatic PCSK9 secretion.

### Plasma lipid levels of *Surf4*^LKO^ mice

**Fig. 2:**
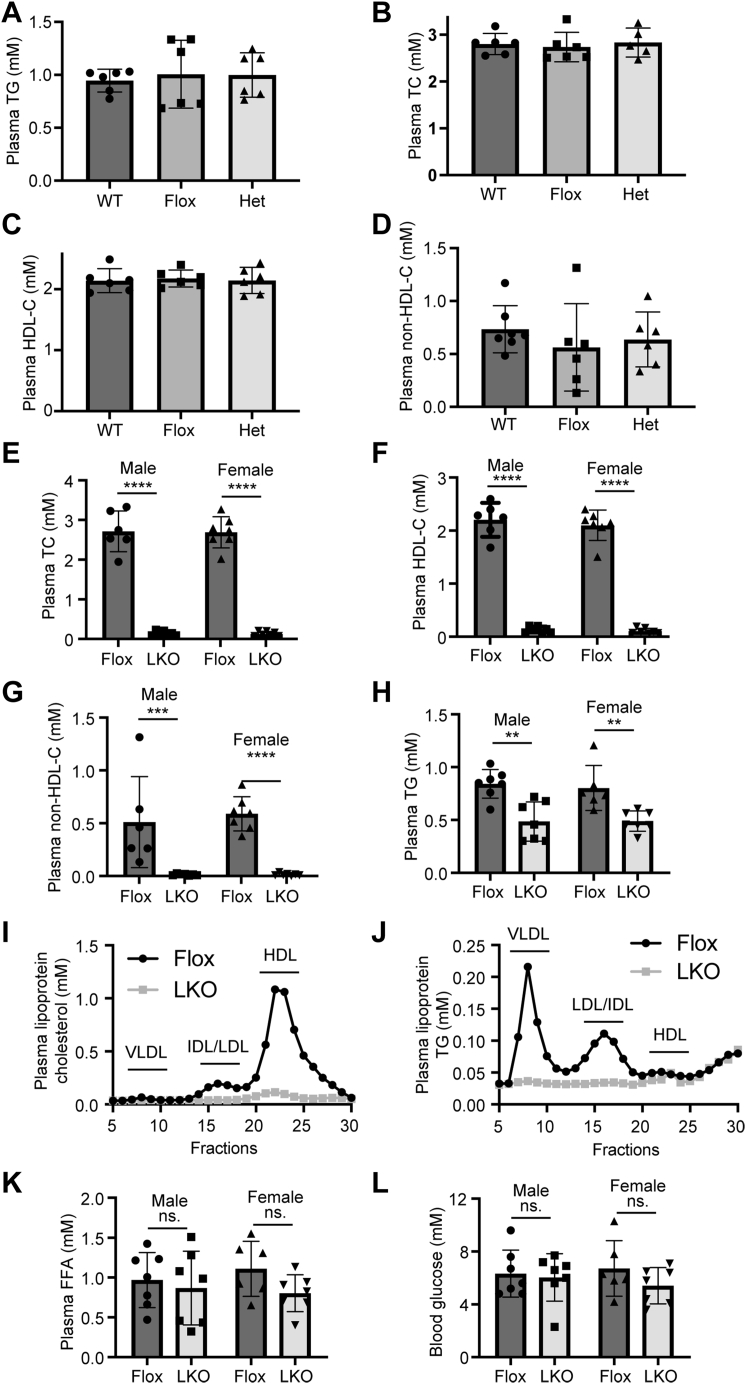
Impact of lacking hepatic Surf4 on plasma lipid and blood glucose levels. A–H: Plasma lipid levels. About 10–14-week-old mice (WT, *Surf4*^Flox^ [Flox], heterozygous *Surf4*^LKO^ [Het], and homozygous *Surf4*^LKO^ [LKO]) on a regular chow diet were fasted for 10 h. Lipids in fasting plasma were measured using their specific enzymatic kits (Applygen) (*n* ≥ 6). Non–HDL-C was calculated by subtracting HDL-C from TC. I and J: Lipid profile. About 100 μl of plasma from each mouse in the same group (*n* = 6) was pooled, applied to fast protein liquid chromatography, and then eluted at 1 ml/fraction. Cholesterol and TG in each fraction were measured using their specific kits (Applygen). K and L: Plasma FFA and blood glucose were measured with an enzymatic kit and a blood glucose meter, respectively (n ≥ 6). Student's *t*-test was used to determine the significant differences between groups. Values of all data were mean ± SD. The significance was defined as ∗*P* < 0.05, ∗∗*P* < 0.01, ∗∗∗*P* < 0.001, and ∗∗∗∗*P* < 0.0001. ns, no significant difference.

We then measured plasma lipid levels and found that the WT, *Surf4*^Flox^, and heterozygous *Surf4*^LKO^ mice showed similar levels of plasma TG, TC, HDL-C, and non-HDL-C (Fig. 2A–D). Conversely, both male and female homozygous *Surf4*^LKO^ mice exhibited a significant reduction in plasma levels of TC, HDL-C, non-HDL-C, and TG compared with *Surf4*^Flox^ mice (Fig. 2E–H). Fast protein liquid chromatography data showed that cholesterol and TG levels were reduced in all fractions, including VLDL, IDL/LDL, and HDL (Fig. 2I, J). On the other hand, plasma-free FAs and blood glucose levels were comparable in *Surf4*^Flox^ and *Surf4*^LKO^ mice (Fig. 2K, L). Therefore, hepatic Surf4 played a vital role in regulating plasma cholesterol and TG levels in mice.

### Effects of Surf4 on TG and apoB secretion

**Fig. 3:**
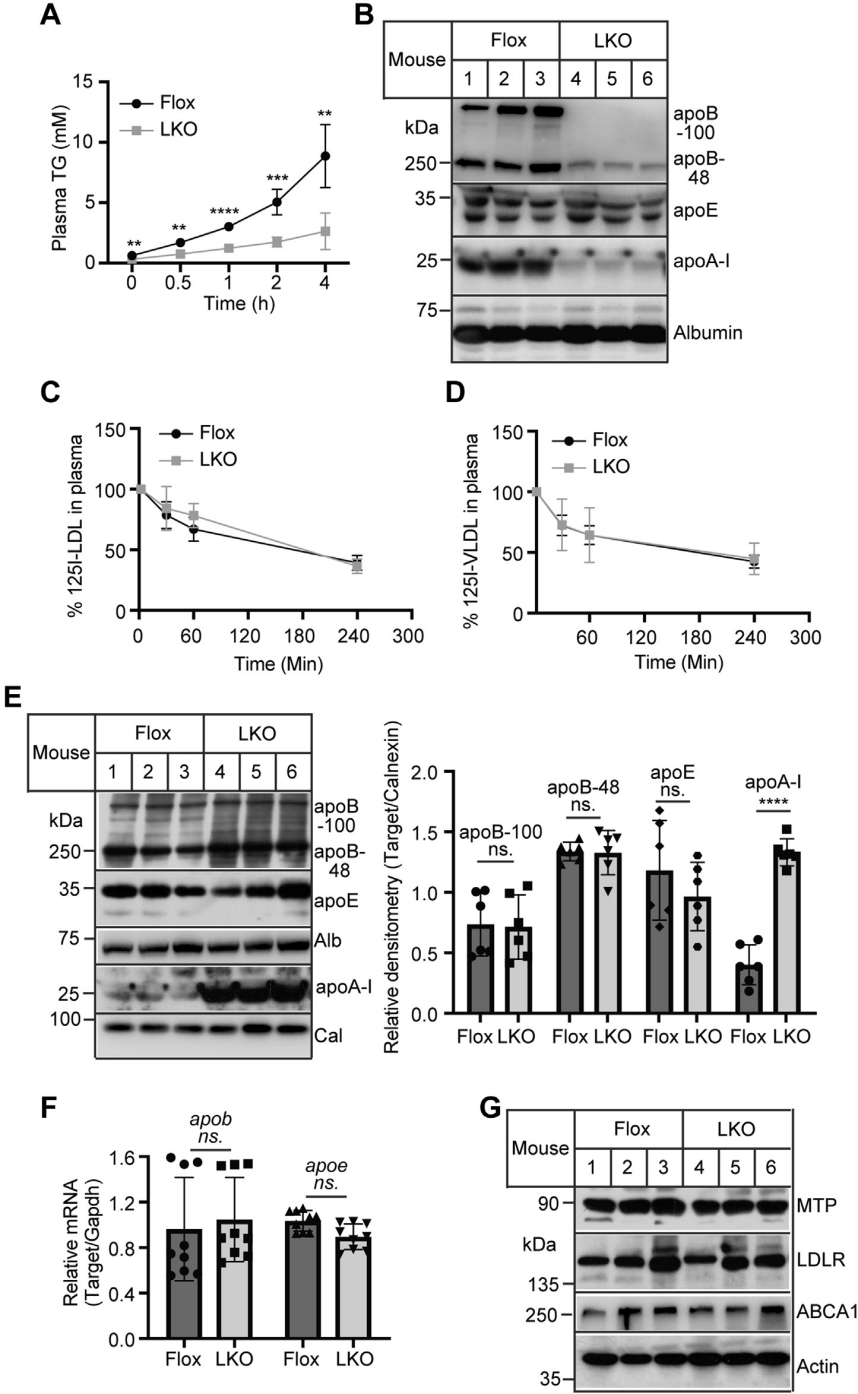
Effects of hepatic Surf4 deficiency on TG secretion and the levels of plasma and liver proteins. A: TG secretion. Mice (12–14-week-old mice, fasted for 10 h, *n* = 6) were injected with p-407. Blood was collected before and 0.5, 1, 2, and 4 h after injection. Plasma TG was measured using an enzymatic kit. B: Immunoblotting (*n* = 6). Different amounts of plasma samples were used to detect apoB, apoE, apoA-I, and Alb, but the same amount of plasma samples from each mice was used to detect each protein in Western blot. Representative images were shown. C and D: Clearance of LDL and VLDL. *Surf4*^Flox^ and *Surf4*^LKO^ mice (*n* = 6) fed a regular chow diet were fasted for 10 h and then administered with ^125^I-LDL (C) or ^125^I-VLDL (D). Blood samples were collected at the indicated time and then subjected to isopropanol precipitation, followed by a γ spectrometer. The clearance was presented as the percentage of radioactivity at different time points to that at the 2 min time point that was set as 100%. E: Immunoblotting. The same amount of liver homogenate was applied to Western blot using antibodies as described. The relative densitometry was the ratio of the densitometry of the protein indicated to that of calnexin (*n* = 6). Representative images were shown. F: Quantitative real-time PCR. The relative mRNA levels were the ratio of the target's mRNA levels indicated to that of *Gapdh* at the same condition (*n* = 9). G: Immunoblotting. The same amount of liver homogenate was applied to Western blot using the indicated antibodies (*n* = 6). Representative images were shown. Student's *t*-test was used to determine the significant differences between groups. Values of all data were mean ± SD. The significance was defined as ∗*P* < 0.05, ∗∗*P* < 0.01, ∗∗∗*P* < 0.001, and ∗∗∗∗*P* < 0.0001. ns, no significant difference.

Next, we dissected how Surf4 affected plasma lipid levels. Fasting plasma TG is mainly contributed by VLDL, a TG-rich lipoprotein produced exclusively by the liver. Thus, we assessed VLDL secretion in *Surf4*^LKO^ and *Surf4*^Flox^ mice and found that plasma TG levels were significantly higher in *Surf4*^Flox^ mice at all time points compared with *Surf4*^LKO^ mice (Fig. 3A). TG levels were markedly increased from 2.922 (1-h time point) to 6.152 (2-h time point) and 10.42 mM (4-h time point) in *Surf4*^Flox^ mice. Conversely, the increase in TG levels during the same period was much less in *Surf4*^LKO^ mice, from 1.329 (1-h time point) to 1.912 (2-h time point) and 3.0 mM (4-h time point). Plasma levels of apoB100 and apoB48 but not apoE or Alb were significantly reduced in *Surf4*^LKO^ mice (Fig. 3B). Plasma apoA-I, the main structural apolipoprotein on HDL, was also significantly reduced in *Surf4*^LKO^ mice. This was consistent with the findings of previous studies on apoB and MTP. Inhibition of hepatic MTP and apoB impaired VLDL assembly and secretion and reduced plasma levels of HDL-C and apoA-I (6, 7, 8, 35). On the other hand, the clearance of LDL and VLDL was comparable in *Surf4*^Flox^ and *Surf4*^LKO^ mice (Fig. 3C, D). Furthermore, we found that the loss of hepatic Surf4 did not significantly affect the protein levels of apoB100, apoB48, apoE, and Alb but increased apoA-I levels in the liver (Fig. 3E). The mRNA levels of apoB and apoE were also comparable in the liver of *Surf4*^Flox^ and *Surf4*^LKO^mice (Fig. 3F). MTP mediates cotranslational lipidation of apoB100. Lacking a functional MTP leads to virtually undetectable plasma apoB100. In addition, LDLR can promote underlipidated apoB100 degradation, reducing VLDL secretion (36, 37). ABCA1 is essential for HDL biogenesis. Thus, we examined the levels of these proteins in liver homogenate and found that the absence of hepatic Surf4 did not alter the expression of LDLR, MTP, and ABCA1 (Fig. 3G).

**Fig. 4:**
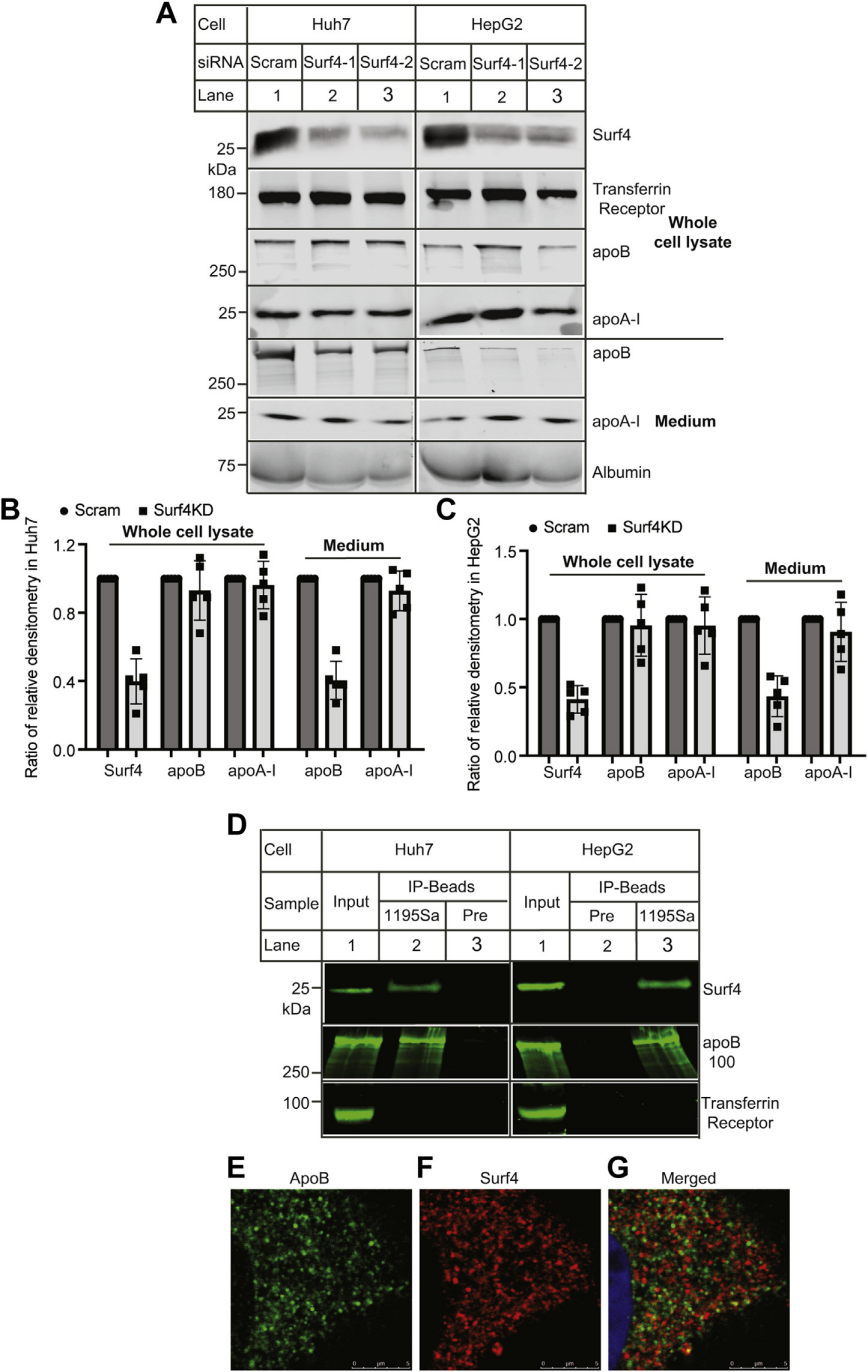
The effect of Surf4 knockdown on the level of apoB and apoA-I in cultured cells. A–C: Knockdown of Surf4. Huh7 and HepG2 cells were transfected with scrambled (Scram.) or one of the two Surf4 siRNAs (Surf4-1 and Surf4-2). About 36 h after transfection, the cells were incubated with oleic acid for 4 h, washed, and then incubated in DMEM without FBS and oleic acid for 16 h. Whole cell lysate was prepared. Same amount of total proteins in whole cell lysate (top) and culture medium (bottom) was applied to Western blot using antibodies indicated. Similar results were obtained from at least five experiments. The relative densitometry was the ratio of the densitometry of target to that of transferrin receptor in whole cell lysate or albumin in medium in the same condition. The ratio of the relative densitometry was the ratio of the relative densitometry of the target in Surf4 knockdown cells to that of the same target in the control cells transfected with scrambled siRNA. The relative densitometry of the target in the control cells was defined as 1. D: Coimmunoprecipitation. The same amount of whole cell lysate from Huh7 or HepG2 was incubated with the preimmune serum (Pre) or a rabbit anti-Surf4 antibody (1195Sa) and protein-G agarose. Whole cell lysate (input) and immunoprecipitated proteins (IP-Beads) were applied to Western blot with antibodies indicated. Similar results were obtained from at least three more experiments. E–G: Confocal microscopy. Huh7 cells were fixed, permeabilized, and then incubated with a goat anti-apoB (abcam) and a rabbit anti-Surf4 antibody, followed by confocal microscopy; apoB (green), Surf4 (red), and DAPI (blue). An *x*–*y* optical section of the cells illustrates the cellular distribution of proteins (magnification: 325×). Representative images were shown.

Surf4 is a cargo receptor located in the ER exit site (17). Was it possible that Surf4 directly mediated apoB and/or apoA-I secretion? To test this hypothesis, we knocked down the expression of Surf4 in human hepatoma-derived cell lines, Huh7 and HepG2 that express and secrete both apoB and apoA-I. Two Surf4 siRNAs efficiently reduced Surf4 expression in whole cell lysate (Fig. 4A, top, lanes 2 and 3 vs. 1; Fig. 4B, C). The levels of apoB100 and apoA-I in whole cell lysate were comparable in the control and Surf4 knockdown cells. Conversely, knockdown of Surf4 reduced the levels of apoB100 but not apoA-I or Alb in culture medium (Fig. 4A, bottom, lanes 2 and 3 vs. 1; Fig. 4B, C). The finding on apoA-I was different from the results of *Surf4*^LKO^ mice, which showed a marked reduction in plasma levels of apoA-I. One possible explanation was that the expression of Surf4 was reduced about 60% in both Huh7 and HepG2 cells (Fig. 4B, C), whereas Surf4 was essentially undetectable in hepatocytes of *Surf4*^LKO^mice (Fig. 1B). The remaining Surf4 in the knockdown cells might be sufficient to mediate apoA-I secretion. We then examined if Surf4 interacted with apoB100 using coimmunoprecipitation. As shown in Fig. 4D, an anti-Surf4 antibody (1195Sa) but not the preimmune serum (Pre) effectively pulled down Surf4 from whole cell lysate isolated from Huh7 (lane 2 vs. 3) and HepG2 cells (lane 3 vs. 2). apoB100 was present only in the Surf4-IP sample, whereas transferrin receptor was undetectable in all immunoprecipitated samples, suggesting an interaction between Surf4 and apoB100. This finding was consistent with a previous report that Surf4 interacted with apoB and facilitated its secretion in HepG2 cells (17). To further confirm this finding, we performed confocal microscopy in Huh7 cells using the anti-Surf4 polyclonal antibody, 1195Sa, that could specifically detect Surf4 (21). We observed that apoB and Surf4 exhibited a similar distribution pattern, residing primarily in the perinuclear region (Fig. 4E, F), where the two proteins were partially colocalized as shown in the merged panel (Fig. 4G, yellow). Therefore, Surf4 appeared to associate with apoB. Taken together, these findings suggest that the lack of hepatic Surf4 may impair VLDL secretion.

### Effects of Surf4 deficiency on hepatic lipids

**Fig. 5:**
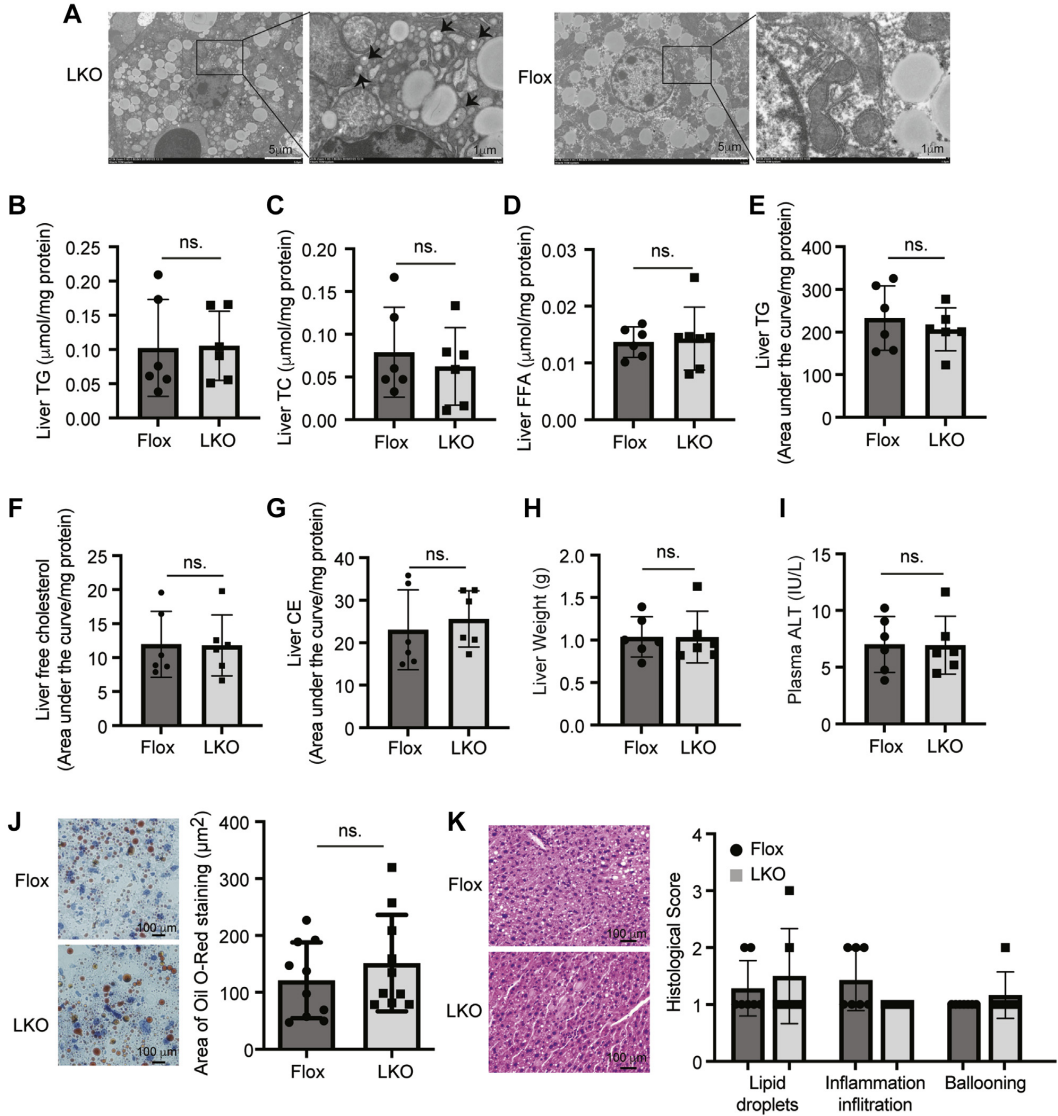
Effect of hepatic Surf4 silencing on liver lipids. About 12–14-week-old male *Surf4*^Flox^ and *Surf4*^LKO^ mice on a regular chow diet were fasted for 10 h before euthanasia. A: Electron microscopy of liver samples from male *Surf4*^Flox^ and *Surf4*^LKO^ mice (*n* = 3). Representative images were shown. B–D: Liver lipids. Lipids were extracted from liver homogenate and then subjected to measurement of TG (B), TC (C), and FFA (D) using their specific enzymatic kits (Nanjing Jiancheng Bioengineering Institute) (*n* = 6). E–G: LC-MS/MS. Lipids were extracted from the liver using the methyl-tert-butyl ether method and applied to LC-MS/MS (*n* = 6). Relative quantification of lipids was calculated by dividing the area under the curve of each substance by that of the internal standard and then normalized to the protein concentrations. H: Weight of a whole liver (*n* = 6). I: Plasma ALT. ALT was measured according to the manufacturer's instruction (Nanjing Jiancheng Bioengineering Institute) (*n* = 6). J and K: Oil Red O (J) and H&E staining (K) of liver sections. The images were quantified using ImageJ (J) (10 mice per group). The slides of H&E staining were assessed for lipid droplets, inflammation infiltration, and ballooning blindly (K) (*n* ≥ 6). Representative figures were shown. Student's *t*-test was used to determine the significant differences between groups. Values of all data were mean ± SD. The significance was defined as ∗*P* < 0.05, ∗∗*P* < 0.01, ∗∗∗*P* < 0.001, and ∗∗∗∗*P* < 0.0001. ns, no significant difference.

Our next experiments were to investigate whether impaired VLDL secretion caused lipid accumulation in the liver of *Surf4*^LKO^ mice. We observed small vesicles in the ER lumen of hepatocytes of *Surf4*^LKO^ mice but not *Surf4*^Flox^ mice (Fig. 5A). These vesicles could be small lipid droplets and/or lipoprotein particles. However, both *Surf4*^Flox^ and *Surf4*^LKO^ mice showed similar hepatic TG, TC, and FFA levels (Fig. 5B–D). The relative levels of TG, free cholesterol, and CE were also comparable in the liver of *Surf4*^LKO^ and *Surf4*^Flox^ mice as measured by LC-MS/MS (Fig. 5E–G). Consistently, knockout of hepatic Surf4 did not significantly alter liver weight or plasma ALT activity (Fig. 5H, I). In addition, Oil Red O and H&E staining of liver sections and histological scores of lipid droplets, inflammation infiltration, and ballooning were comparable in *Surf4*^LKO^ and *Surf4*^Flox^ mice (Fig. 5J, K). Thus, lacking hepatic Surf4 did not cause hepatic lipid accumulation or significant liver damage.

**Fig. 6:**
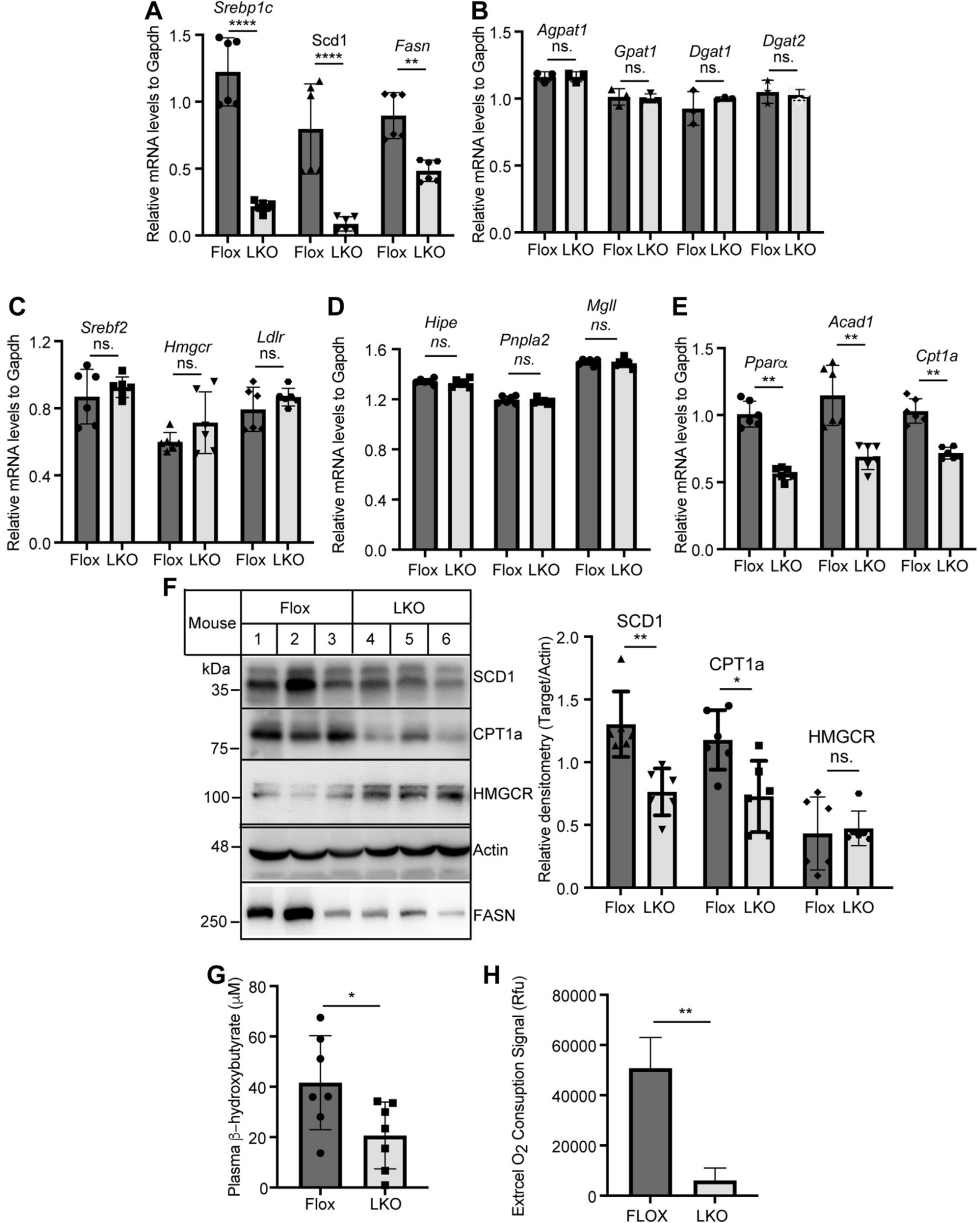
Effect of hepatic Surf4 knockout on gene expression. Mice were treated as described in the legend to Fig. 3. A–E: Quantitative real-time PCR (six mice per group). The relative mRNA levels were the ratio of the mRNA level of the target to that of *Gapdh* at the same condition. F: Western blot of liver homogenate with antibodies indicated. The relative densitometry was the ratio of the densitometry of the protein indicated to that of actin (*n* = 6). Representative images were shown. G: Plasma ketone bodies were measured using a kit (Nanjing Jiancheng Bioengineering Institute) (*n* = 7). H: FA oxidation. Primary hepatocytes isolated from *Surf4*^Flox^ or *Surf4*^LKO^ mice were subjected to measurement of FA oxidation using a commercial kit from abcam. Student's *t*-test was used to determine the significant differences between groups. Values of all data were mean ± SD. The significance was defined as ∗*P* < 0.05, ∗∗*P* < 0.01, ∗∗∗*P* < 0.001, and ∗∗∗∗*P* < 0.0001. *P* > 0.05, no significant difference (ns).

We then examined mRNA levels of the genes involved in lipid metabolism in the liver. As shown in Fig. 6A, mRNA levels of genes encoding proteins important for de novo lipogenesis, including *Srebp1c*, *Scd1*, and *Fasn*, were significantly reduced in *Surf4*^LKO^ mice. In contrast, the absence of hepatic Surf4 did not significantly change the mRNA levels of genes encoding important factors for TG synthesis (*Agpat1*, *Gpat1*, *Dgat1*, *and Dgat2*), cholesterol metabolism (*Srebf2*, *Hmgcr*, and *Ldlr*), and lipolysis (*Hipe*, *Pnpla2*, and *Mgll*) (Fig. 6B–D). Interestingly, *Surf4*^LKO^ mice also exhibited a significant reduction in the mRNA levels of genes involved in FA β-oxidation (*Pparα*, *Acad1*, *and Cpt1a*) (Fig. 6E). Consistently, the protein levels of stearoyl-CoA desaturase-1 (SCD1), carnitine palmitoyltransferase 1a, and fatty acid synthase were significantly decreased (Fig. 6F). Plasma levels of ketone body, β-hydroxybutyrate, in *Surf4*^LKO^ mice were reduced (Fig. 6G). FA oxidation was also significantly reduced in primary hepatocytes isolated from *Surf4*^LKO^ mice (Fig. 6H). Taken together, these findings indicated that FA oxidation was reduced in the liver of *Surf4*^LKO^ mice and suggested that lacking hepatic Surf4 might reduce de novo lipogenesis.

### Effects of Surf4 on the development of atherosclerosis

**Fig. 7:**
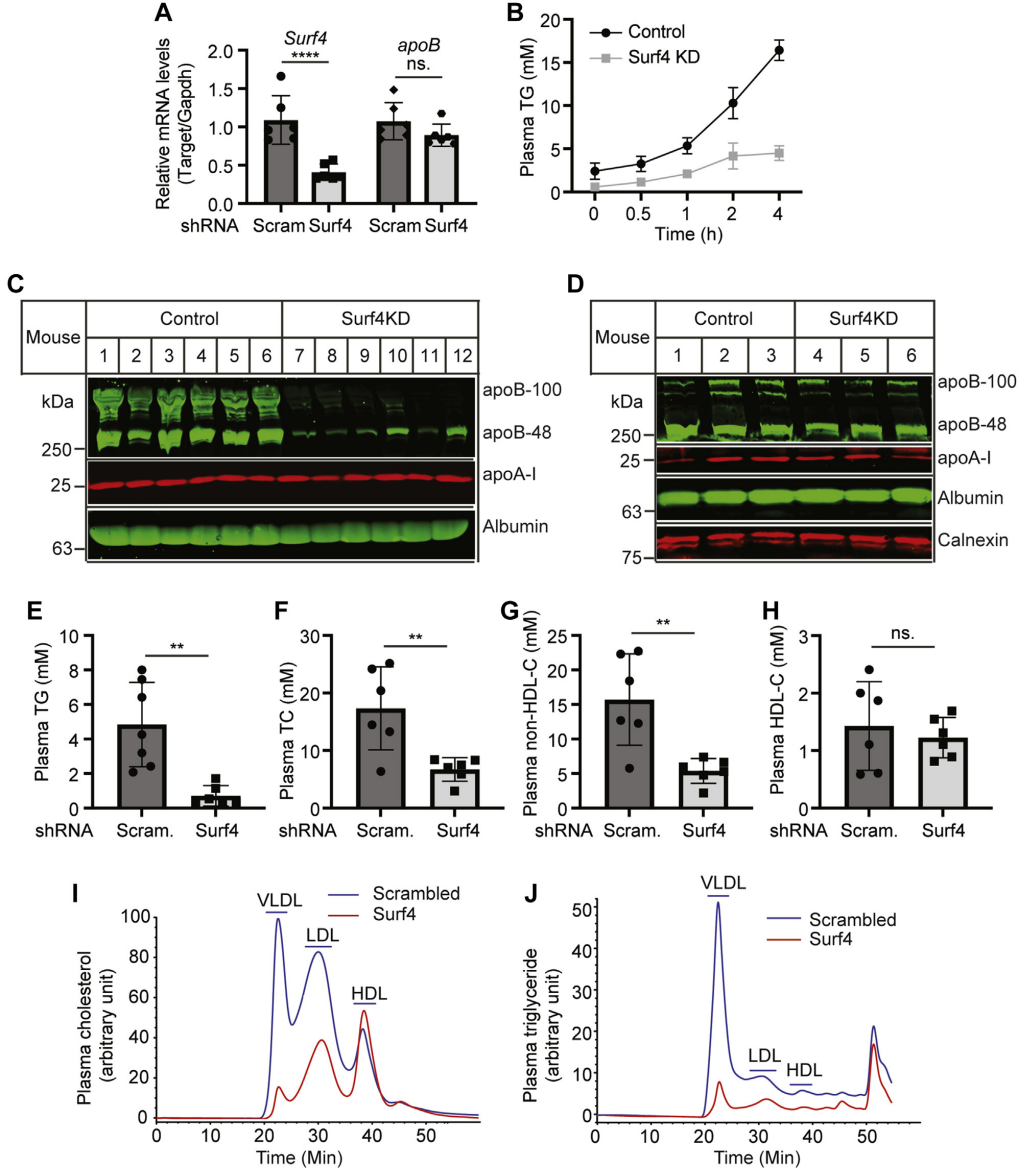
The effect of Surf4 knockdown on plasma lipid levels and the expression of apoB and apoA-I in *Ldlr*^−/−^ mice. About 8–10-week-old male *Ldlr*^−/−^ mice were injected with AAV-scrambled (Scram.) or Surf4 shRNA and then fed the Western-type diet for 14 weeks. A: Quantitative real-time PCR. The relative mRNA levels were the ratio of the mRNA levels of the target gene to that of *Gapdh* (*n* = 6). B: TG secretion. Blood was collected from mice before and 0.5, 1, 2, and 4 h after P-407 injection. Plasma TG was measured using a kit (Wako Diagnostics) (*n* = 4). C and D: Immunoblotting of the same amount of plasma (C) or liver homogenate (D) with antibodies indicated (*n* = 6). Representative images were shown. E–H: Plasma lipids were measured using kits (Wako Diagnostics) (*n* = 6). I and J: Lipid profile. About 5 μl of plasma from each mouse in the same group (*n* = 6) was pooled and applied to fast protein liquid chromatography analysis of cholesterol (I) and TG (J). Student's *t*-test was used to determine the significant differences between groups. Values of all data were mean ± SD. The significance was defined as ∗*P* < 0.05, ∗∗*P* < 0.01, ∗∗∗*P* < 0.001, and ∗∗∗∗*P* < 0.0001. *P* > 0.05, no significant difference (ns).

**Fig. 8:**
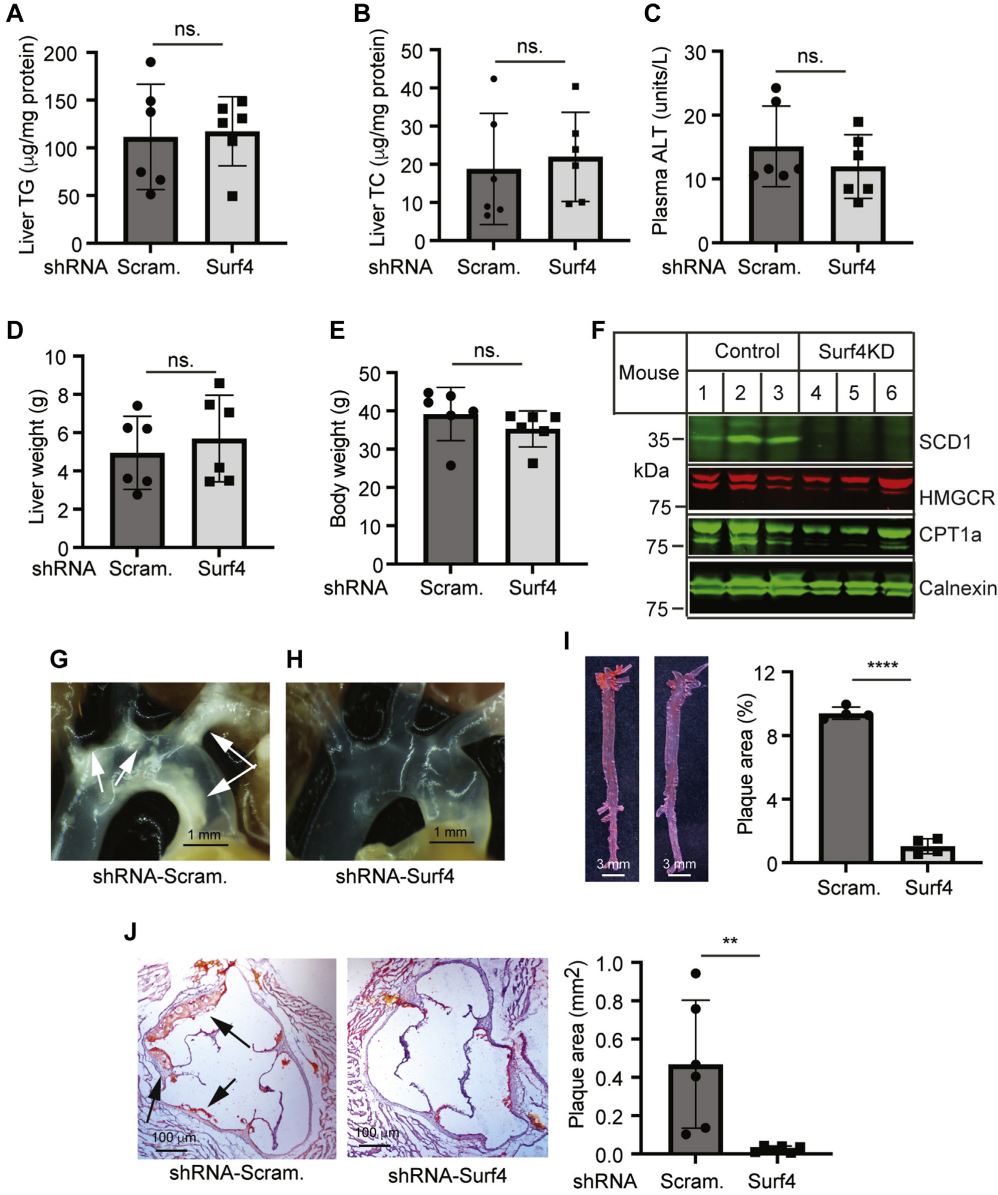
The effect of Surf4 knockdown on the levels of liver lipids and the development of atherosclerosis in *Ldlr*^−/−^ mice. Mice were treated as described in the legend to Fig. 7. A and B: Liver lipids were extracted from mouse livers for the measurement of TG (A) and TC (B) using enzymatic kits (Wako Diagnostics) (six mice per group). C: Plasma ALT measured with a kit (Cayman Chemical Company). D and E: Liver and body weight of mice (six mice per group). F: Immunoblotting of the same amount of liver homogenate from the control and Surf4 knockdown mice (six mice per group) with antibodies indicated. Representative images were shown. G–H: Representative images of aortic arches. Similar results were observed in five more mice in each group. I and J: Oil Red O staining of the aorta (I, four mice per group) and heart sections (J, six mice per group). Magnification, 40×. Atherosclerotic lesions in the aorta and aortic sinus were quantified using OMAX ToupView. Representative figures were shown. Student's *t*-test was used to determine the significant differences between groups. Values of all data were mean ± SD. The significance was defined as ∗*P* < 0.05, ∗∗*P* < 0.01, ∗∗∗*P* < 0.001, and ∗∗∗∗*P* < 0.0001. *P* > 0.05, no significant difference (ns).

Next, we investigated the effect of lacking hepatic Surf4 on the development of atherosclerosis in *Ldlr*^−/−^ mice. Male *Ldlr*^−/−^ mice were injected with AAV-scrambled or Surf4 shRNA and then fed the Western-type diet for 14 weeks. As shown in Fig. 7A, AAV-Surf4 shRNA efficiently reduced *Surf4* mRNA levels by approximately 61% but did not affect the mRNA level of *apoB* in mouse liver. *Surf4* silencing markedly reduced TG secretion (Fig. 7B) and plasma levels of apoB100 and apoB48 but not apoA-I (Fig. 7C). On the other hand, the levels of apoB100, apoB48, and apoA-I were comparable in liver homogenate of the control and Surf4 knockdown mice (Fig. 7D). Plasma levels of TG, TC, non-HDL-C, but not HDL-C, were significantly reduced in Surf4 knockdown mice (Fig. 7E–H). Consistently, fast protein liquid chromatography data showed that cholesterol levels were dramatically reduced in both VLDL and LDL fractions, but not HDL of Surf4 knockdown mice (Fig. 7I). Surf4 silencing also reduced plasma TG levels in both VLDL and LDL fractions (Fig. 7J). We then examined if Surf4 knockdown caused liver damage in *Ldlr*^−/−^ mice. As shown in Fig. 8A–E, the levels of liver TG and TC, plasma ALT activity, liver, and body weight were comparable in the control and Surf4 knockdown mice. Western blot analysis revealed that knockdown of Surf4 reduced the protein levels of SCD1 but not HMGCR or carnitine palmitoyltransferase 1a (Fig. 8F). Thus, knockdown of Surf4 in *Ldlr*^−/−^ mice significantly reduced plasma levels of cholesterol and TG but did not cause hepatic lipid accumulation or significant liver damage.

Next, we examined the effect of Surf4 silencing on the development of atherosclerosis and found that *Ldlr*^−/−^ mice injected with AAV-scrambled shRNA formed plaques in the aortic arch, which were virtually eliminated in *Surf4* knockdown mice (Fig. 8G, H). Oil Red O staining of the aorta consistently showed a significant reduction in lesion area in the aorta of Surf4 knockdown mice (Fig. 8I; 9.4% in the control and 1% in the Surf4 knockdown group, *P* < 0.0001). Average atherosclerotic lesion areas in the aortic sinus were also dramatically reduced in mice injected with AAV-Surf4 shRNA (0.4683 and 0.02594 mm^2^ in AAV-Scrambled and Surf4 shRNA-injected mice, respectively, *P* = 0.0089) (Fig. 8J). Thus, knockdown of Surf4 markedly reduced the development of atherosclerosis *in Ldlr*^−/−^ mice.

## Discussion

VLDL, a TG-rich lipoprotein exclusively produced in and secreted from the liver, is catabolized to LDL in the circulatory system. Inhibition of VLDL secretion can significantly reduce plasma levels of LDL-C and alleviate the development of atherosclerosis. VLDL is transported from the ER to the Golgi apparatus through VTV(1). However, how VLDL is recruited from the ER lumen to VTV is unclear. Our findings indicate that Surf4 may recruit VLDL from the ER lumen to VTV for the subsequent delivery to the Golgi apparatus. However, Surf4 was not required for PCSK9 secretion in vivo.

When this article was under preparation, Wang *et al.* (38) reported that a variance in Surf4, rs3758348, was significantly associated with reduced plasma levels of TC and LDL-C in humans. Consistently, they reported that knockout of hepatic Surf4 using CRISPR-Cas9 significantly reduced plasma levels of cholesterol and TGs and reduced the development of atherosclerosis in a mouse model of hypercholesterolemia induced by overexpressing PCSK9. We and Wang *et al.* both found that VLDL was trapped in the ER lumen of hepatocytes of Surf4 knockout mice, while plasma ALT levels were comparable in the control and Surf4 silencing mice. However, there were several differences between our findings and theirs. First, we found that the lack of hepatic Surf4 did not cause TG and cholesterol accumulation in the liver. We confirmed these findings using various methods: oil-red O staining and measurement of liver lipids using enzymatic kits and LC-MS/MS. Conversely, Wang *et al.* reported that knockdown of Surf4 in their mouse model significantly increased liver TG and cholesterol levels. They also knocked down Surf4 expression by administering AAV-TBG-Cre to *Surf4*^Flox^ mice and reported that liver TG but not cholesterol levels were increased in the Surf4 knockdown mice. Second, Wang *et al.* reported that plasma cholesterol levels were reduced in heterozygous Surf4 liver knockout mice. However, we observed that haplodeficiency of hepatic Surf4 did not significantly affect plasma levels of cholesterol and TGs. Our findings were consistent with a recent report that plasma cholesterol and TG levels were comparable in heterozygous Surf4 global knockout mice and the WT mice (39). It is possible that the levels of Surf4 expressed from one copy of the *Surf4* gene are enough to mediate VLDL secretion. Third, Wang *et al.* claimed that Surf4 mediated secretion of lipoproteins apoA-I and apoB. We also observed that plasma levels of apoA-I and HDL were significantly reduced in *Surf4*^LKO^ mice. However, knockdown of Surf4 in cultured human hepatocytes did not affect apoA-I secretion. Plasma apoA-I and HDL-C levels were also comparable in the control and Surf4 knockdown *Ldlr*^−/−^ mice. The reasons for these differences were unclear. Different approaches were used in the two studies to silence Surf4 expression in mouse liver. We bred *Surf4*^*Flox*^ mice with Alb-Cre mice to delete functional Surf4 specifically in hepatocytes. Surf4 was knocked out perinatally. Wang *et al.* administered Cre-dependent spCas9 knock-in mice with AAV-Surf4 gRNAs to knock down Surf4 expression in the liver of adult mice. It is also possible that the knockdown efficiency of Surf4 might be different in the two studies, causing the different phenotypes.

The reduction in fasting plasma levels of LDL-C and apoB100 was most likely caused by impaired VLDL secretion. The exact mechanism by which plasma HDL-C levels were reduced is unclear. In *Surf4*^LKO^ mice, plasma HDL-C (Fig. 2F) and apoA-I levels (Fig. 3B) were significantly reduced, whereas liver apoA-I levels were increased (Fig. 3E). These findings were consistent with the report from Wang *et al.* (38) and indicated that Surf4 might also mediate apoA-I secretion. However, knockdown of Surf4 in Huh7 and HepG2 cells did not significantly affect the level of apoA-I in whole cell lysate and culture medium (Fig. 4A–C). Similarly, knockdown of Surf4 expression in *Ldlr*^−/−^ mice did not significantly affect the level of apoA-I in plasma (Fig. 7C) and liver homogenate (Fig. 7D). Knockdown of Surf4 in *Ldlr*^−/−^ mice also did not affect plasma levels of HDL-C even though non-HDL-C levels were markedly reduced (Fig. 7G–I). One main difference between *Surf4*^LKO^ mice and the knockdown experiments was that hepatic Surf4 was essentially undetectable in *Surf4*^LKO^ mice (Fig. 1B), whereas the expression of Surf4 was only partially reduced in cultured cells and *Ldlr*^−/−^ mice (Figs. 4A–C and 7A). Similarly, the Surf4 variant, rs3758348 that reduced Surf4 expression, significantly reduced plasma TC and LDL-C but not HDL-C levels in humans (38). Thus, the remaining Surf4 in the knockdown experiments might still be able to facilitate secretion of apoA-I effectively.

Hepatic TG homeostasis is regulated by de novo lipogenesis, FA uptake, FA oxidation, and VLDL secretion. Inhibition of apoB and MTP impaired VLDL production and increased hepatic TG, leading to liver steatosis (8, 40, 41, 42). On the other hand, Conlon *et al.* (9) reported that knockdown of apoB for 6 weeks did not cause liver steatosis even though a mild lipid accumulation was observed at 3 weeks after apoB knockdown. Similarly, our findings showed that VLDL secretion was impaired and FA oxidation was reduced in *Surf4*^LKO^ mice, and yet we did not observe significant hepatic TG accumulation or notable liver damage. It has been reported that de novo lipogenesis was a key contributor to fatty liver and increased significantly in subjects with nonalcoholic fatty liver disease (43). The expression of SCD1, a rate-limiting enzyme in converting saturated FAs to monounsaturated FAs in de novo lipogenesis, was significantly reduced in the liver of *Surf4*^LKO^ mice and Surf4 knockdown *Ldlr*^−/−^ mice. This finding indicated a reduction in de novo lipogenesis, which might partially explain why liver TG levels were not increased in *Surf4*^LKO^ mice. It also explained the reduction in the expression of *PPAR-α* target genes and plasma levels of ketone bodies because de novo lipogenesis provides endogenous ligands for activating PPAR-α transcriptional activity to upregulate expression of genes involved in FA β-oxidation (44). Of note, de novo lipogenesis is an important contributor to fatty liver in hyperinsulinemia and insulin resistance but not to hepatic TG levels under normal physiological conditions (45). Conlon *et al.* (9) reported that knockdown of apoB100 in mice caused accumulation of lipid droplets in the ER lumen of hepatocytes, which triggered ER autophagy, effectively removing TG accumulated in mouse hepatocytes. Similarly, we observed small vesicles in the ER lumen of hepatocytes in *Surf4*^LKO^ mice (Fig. 5A). Furthermore, it has been reported that apoB accumulated in the ER lumen of hepatocytes increased autophagy, leading to resolution (46). The levels of plasma apoB were markedly reduced in *Surf4*^LKO^ mice (Fig. 3B); however, we did not observe a significant increase in hepatic apoB levels (Fig. 3E). Therefore, autophagy may play a role in clearing apoB and TG retained in the liver of *Surf4*^LKO^ mice. The lack of hepatic Surf4 might also affect metabolic rate and hepatic FA uptake and phospholipid metabolism. Experiments are undergoing to investigate these possibilities.

In summary, we provided evidence for the physiological role of Surf4, which mediates VLDL secretion. Inhibition of hepatic Surf4 reduced VLDL secretion and the development of atherosclerosis. These findings were consistent with the recent report from the Chen group (38). Wang *et al.* (38) also reported that Surf4 interacted with SAR1B to facilitate the transport of VLDL from the ER to the Golgi. Therefore, secretion of VLDL appears to require the formation of VTVs with the help of COPII, TANGO1, SAR1B, KLH12, and Surf4 as indicated by Ginsberg (47). Interestingly, inhibition of hepatic Surf4 did not cause hepatic lipid accumulation or notable liver damage in mice fed a regular chow diet or *Ldlr*^−/−^ mice fed the Western-type diet, even when VLDL secretion was impaired. Furthermore, despite that HDL was significantly reduced in *Surf4*^LKO^ mice, knockdown of Surf4 expression in cultured human hepatocytes and *Ldlr*^−/−^ mice had no significant effect on apoA-I secretion and plasma HDL-C levels. Thus, our findings indicate that Surf4-based therapy can potentially lower plasma LDL-C levels by inhibiting VLDL secretion and subsequent LDL production, thereby reducing the risk of atherosclerosis for patients who are intolerant to or cannot be effectively managed by existing therapies.

## Data availability

The data underlying this article are available in the article and in its online supplemental data. Additional data underlying this article will be shared on reasonable request to the corresponding author.

## Supplemental data

This article contains supplemental data.

## Conflict of interest

The authors declare that they have no conflicts of interest with the contents of this article.
