# Supplementary material for: Atherosclerosis-associated hepatic secretion of VLDL but not PCSK9 is dependent on cargo receptor protein Surf4
Source: J Lipid Res. 2021 Jun 9;62:100091. doi: 10.1016/j.jlr.2021.100091 (PMC8261665; doi:10.1016/j.jlr.2021.100091)
Supplement: Source data [file mmc1.pdf]

Figure 1

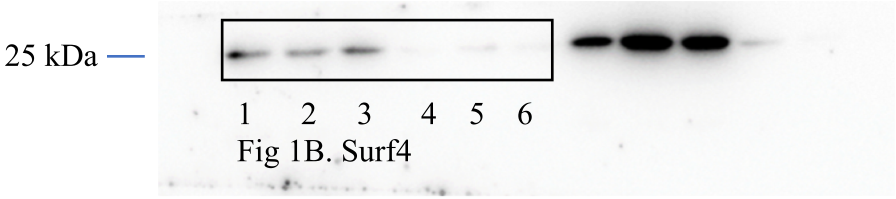

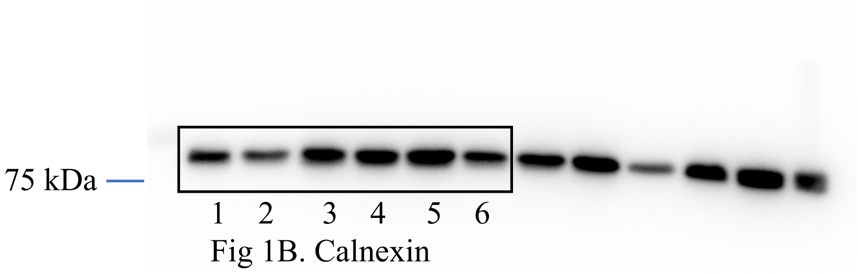

Fig 1B. Surf4. Whole liver lysate was subjected to WB. The membrane was blotted with an anti-Surf4 antibody. Antibody binding was detected using HRP-conjugated secondary antibodies. SDS-PAGE (12%).

Fig 1B. **Calnexin.** Whole liver lysate was subjected to WB. The membrane was blotted with a rabbit anti-calnexin antibody. Antibody binding was detected using HRP-conjugated secondary antibodies. SDS-PAGE (12%).

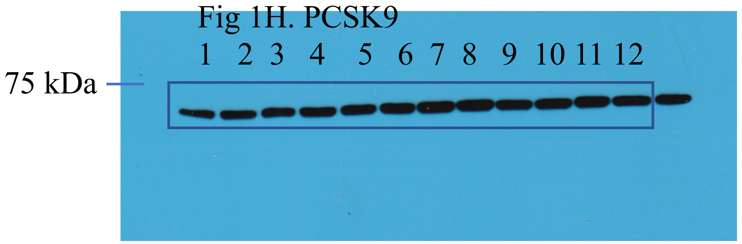

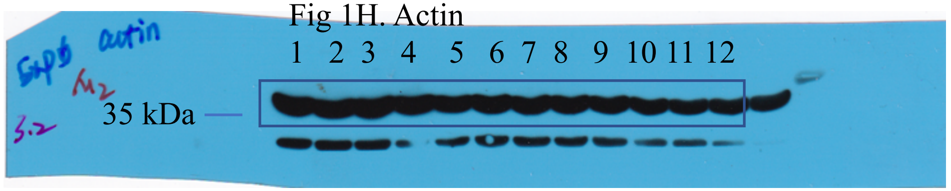

Fig 1H. PCSK9. The membrane was cut into halves. The top part was blotted with a rabbit anti-mouse PCSK9 antibody (abcam). Antibody binding was detected using HRP-conjugated secondary antibodies. SDS-PAGE (10%).

Fig 1H. Actin. The membrane was cut into halves. The bottom part was blotted with a rabbit anti-actin antibody (Bioss). Antibody binding was detected using HRP-conjugated secondary antibodies. SDS-PAGE (10%).

Figure 1 K

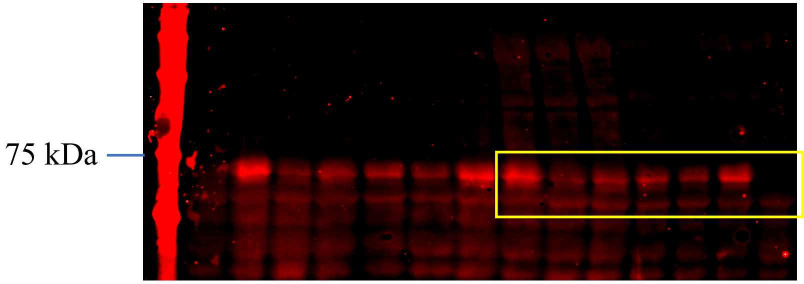

Fig 1K. PCSK9. The membrane was blotted with a rabbit anti-mouse PCSK9 antibody (abcam). Antibody binding was detected using IRDye680-Donkey anti-rabbit IgG. SDS-PAGE (8%).

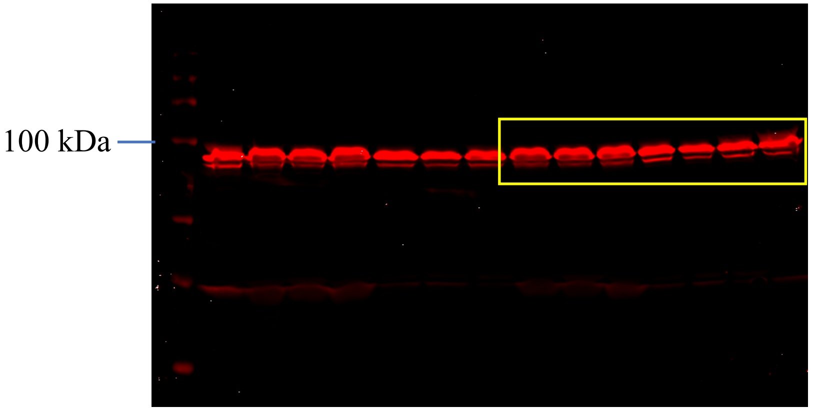

Fig 1K. Calnexin. The membrane was blotted with a rabbit anti-Calnexin antibody (abcam). Antibody binding was detected using IRDye680-Donkey anti-rabbit IgG. SDS-PAGE (80%).

Figure 3B

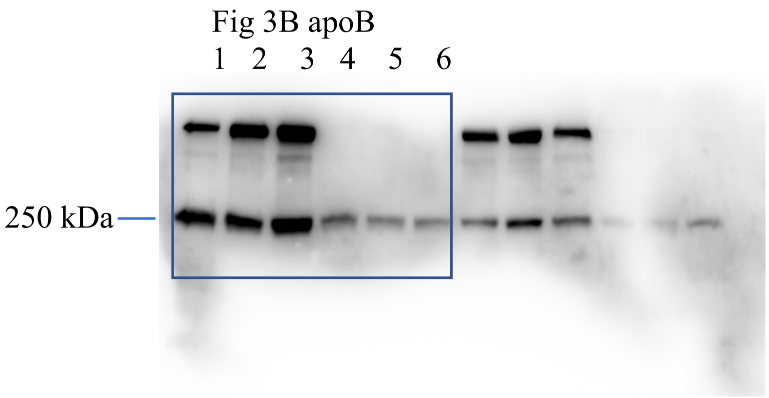

Fig 3B. apoB. The membrane was blotted with a rabbit anti-apoB antibody (abcam). SDS-PAGE (6%). Antibody binding was detected using HRP-conjugated secondary antibodies.

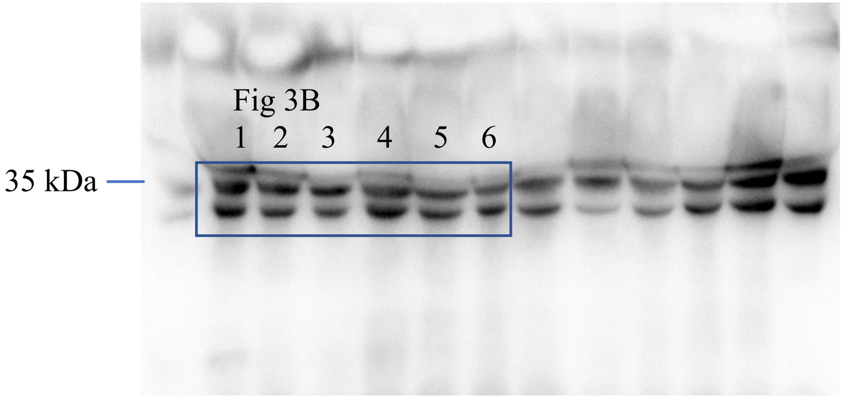

Fig 3B. apoE. The membrane was blotted with a mouse anti-apoE antibody (abcam). SDS-PAGE (10%). Antibody binding was detected using HRP-conjugated secondary antibodies.

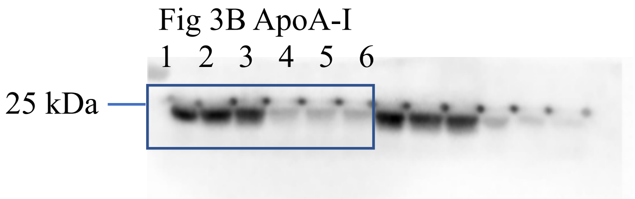

Fig 3B. ApoA-I. The membrane was blotted with a rabbit anti-apoA-I antibody (Bioss). SDS-PAGE (10%). Antibody binding was detected using HRP-conjugated secondary antibodies.

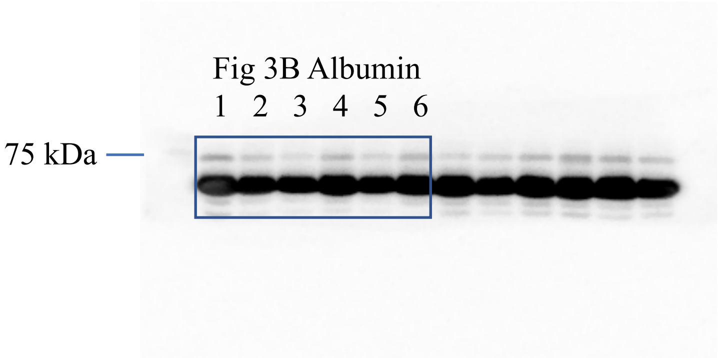

Fig 3B. Albumin. The membrane was blotted with a rabbit anti-albumin antibody (abcam). SDS-PAGE (10%). Antibody binding was detected using HRP-conjugated secondary antibodies.

Figure 3 E

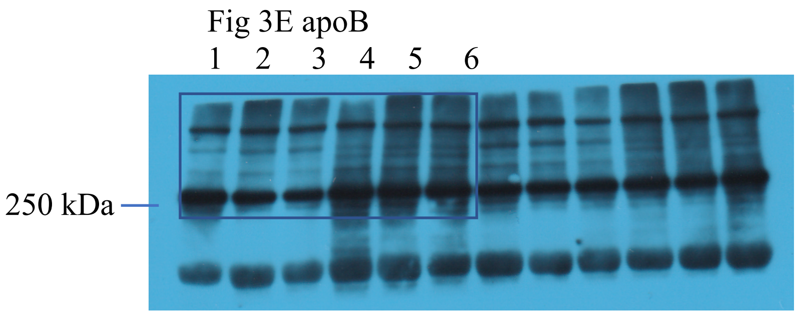

Fig 3E. apoB. The membrane was blotted with a rabbit anti-apoB antibody (abcam). SDS-PAGE (6%). Antibody binding was detected using HRP-conjugated secondary antibodies.

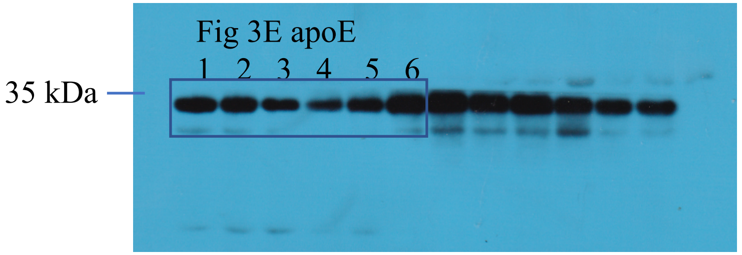

Fig 3E. apoE. The membrane was cut into halves. The bottom halves was blotted with a mouse anti-apoE antibody (abcam). SDS-PAGE (10%). Antibody binding was detected using HRP-conjugated secondary antibodies.

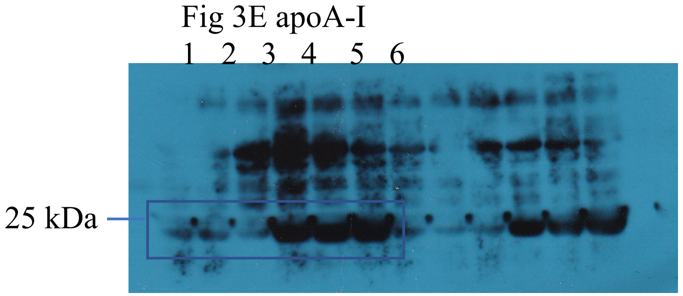

Fig 3E. apoA-I. The membrane was blotted with a mouse anti-apoA-I antibody (abcam). SDS-PAGE (10%). Antibody binding was detected using HRP-conjugated secondary antibodies.

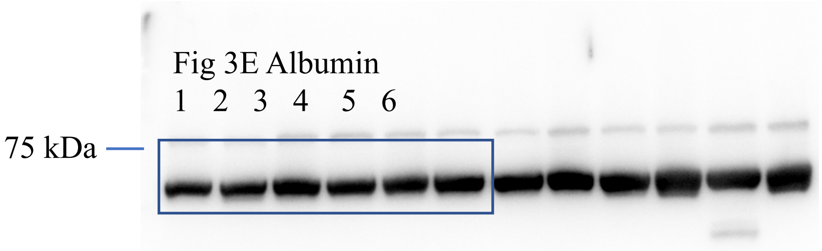

Fig 3E. Albumin. The membrane was blotted with a rabbit anti-albumin antibody (abcam). SDS-PAGE (10%). Antibody binding was detected using HRP-conjugated secondary antibodies.

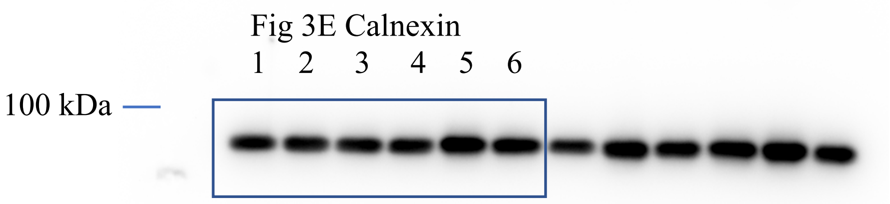

Fig 3E. Calnexin. The membrane was blotted with a rabbit anti-Calnexin antibody (abcam). SDS-PAGE (10%). Antibody binding was detected using HRP-conjugated secondary antibodies.

Figure 3 G

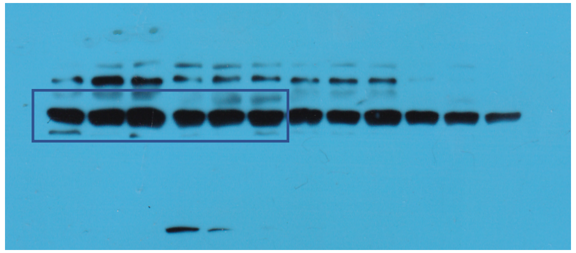

Fig 3G. MTP. The membrane was blotted with a rabbit anti-MTP antibody (Bioworld). SDS-PAGE (8%). Antibody binding was detected using HRP-conjugated secondary antibodies.

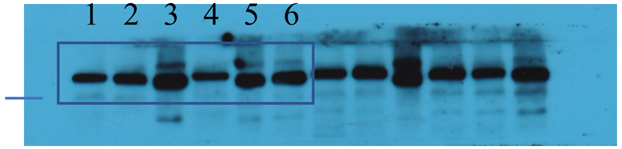

Fig 3G. LDLR. The membrane was cut into halves. The top part was blotted with a rabbit anti-LDLR antibody (abcam). SDS-PAGE (8%). Antibody binding was detected using HRP-conjugated secondary antibodies.

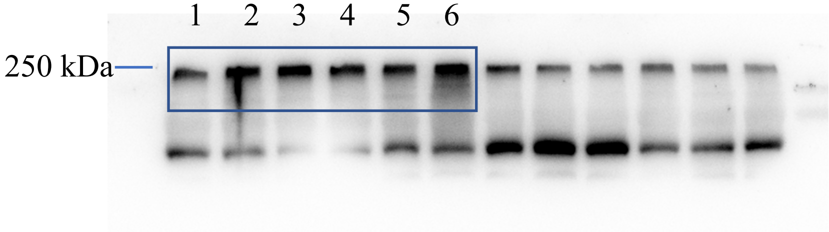

Fig 3G. ABCA1. The membrane was blotted with an anti-ABCA1 antibody. SDS-PAGE (8%). Antibody binding was detected using HRP-conjugated secondary antibodies.

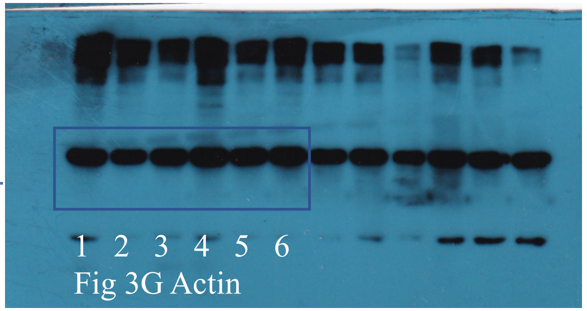

Fig 3G. Actin. The membrane was blotted with a rabbit anti-actin antibody (Bioss). Antibody binding was detected using HRP-conjugated secondary antibodies. SDS-PAGE (10%).

Figure 4 A-whole cell lysate-Huh7

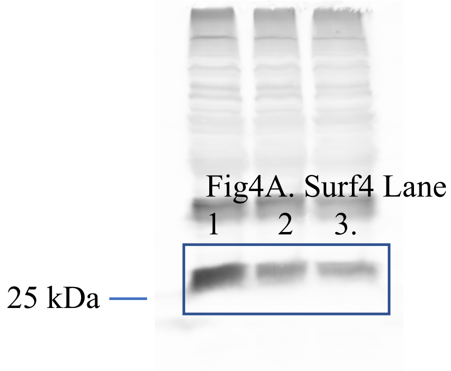

Fig 4A. Huh7 whole cell lysate Surf4. The membrane was blotted with an anti-Surf4 antibody. Antibody binding was detected using IRDye®680 or IRDye®800-labeled donkey anti-mouse, rabbit, or goat IgG (Li-Cor), followed by imaging on a Licor Odyssey Infrared Imaging System (Li-Cor). SDS-PAGE (10%).

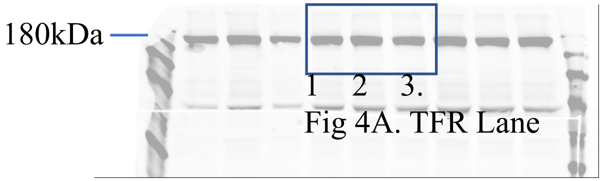

Fig 4A. **Huh7 whole cell lysate TFR**. The membrane was cut into halves. The top part was blotted with a mouse anti-transferrin receptor (TFR) antibody (BD Biosciences). Antibody binding was detected using IRDye®680 or IRDye®800-labeled donkey anti-mouse, rabbit, or goat IgG (Li-Cor), followed by imaging on a Licor Odyssey Infrared Imaging System (Li-Cor). SDS-PAGE (8%).

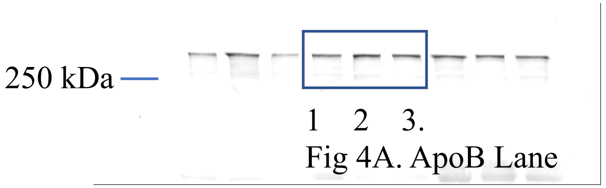

Fig 4A. Huh7 whole cell lysate ApoB. The membrane was cut into halves. The top part was blotted with a goat anti-apoB antibody (Millipore Sigma). Antibody binding was detected using IRDye®680 or IRDye®800-labeled donkey anti-mouse, rabbit, or goat IgG (Li-Cor), followed by imaging on a Licor Odyssey Infrared Imaging System (Li-Cor). SDS-PAGE (6%).

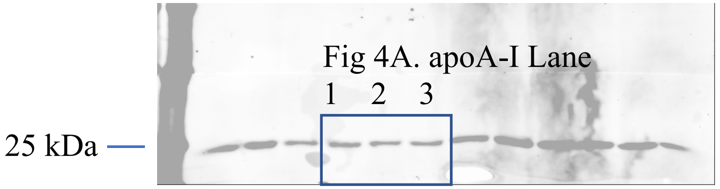

Fig 4A. Huh7 whole cell lysate ApoA-I. The membrane was cut into halves. The bottom part was blotted with a mouse anti-apoA-I antibody (Cell signaling). Antibody binding was detected using IRDye®680 or IRDye®800-labeled donkey anti-mouse, rabbit, or goat IgG (Li-Cor), followed by imaging on a Licor Odyssey Infrared Imaging System (Li-Cor). SDS-PAGE (12%).

Figure 4 A-Culture medium-Huh7

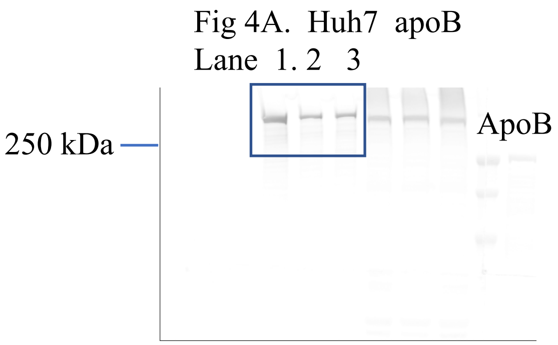

Fig. 4A. Huh7 medium apoB. The membrane was cut into halves. The top part was blotted with a goat-anti apoB antibody (Millipore Sigma). Antibody binding was detected using IRDye®800-labeled donkey anti-goat IgG (Li-Cor), followed by imaging on a Licor Odyssey Infrared Imaging System (Li-Cor). SDS-PAGE (6%).

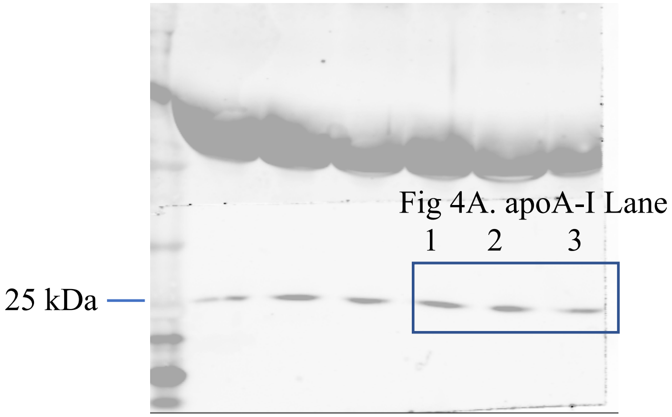

Fig. 4A. Huh7-medium apoA-I. The membrane was cut into halves. The bottom part was blotted with a mouse anti apoA-I antibody (Cell signaling). Antibody binding was detected using IRDye®680-labeled donkey anti-mouse IgG (Li-Cor), followed by imaging on a Licor Odyssey Infrared Imaging System (Li-Cor). SDS-PAGE (12%).

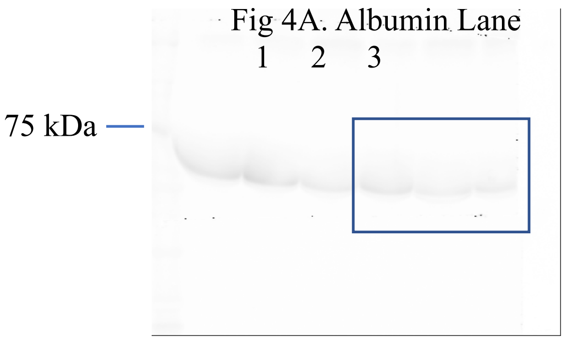

Fig. 4A Huh7 medium albumin. The membrane was cut into halves. The top part was blotted with a rabbit anti-albumin antibody (abcam). Antibody binding was detected using IRDye®680-labeled donkey anti-rabbit IgG (Li-Cor), followed by imaging on a Licor Odyssey Infrared Imaging System (Li-Cor). SDS-PAGE (12%).

Figure 4 A-whole cell lysate-HepG2

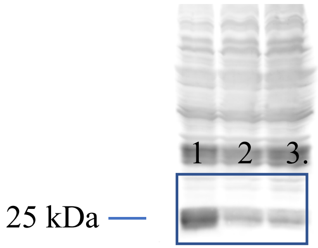

Fig. 4A. HepG2 whole cell lysate Surf4. The membrane was blotted with an anti-Surf4 antibody. Antibody binding was detected using IRDye®680 or IRDye®800-labeled donkey anti-mouse, rabbit, or goat IgG (Li-Cor), followed by imaging on a Licor Odyssey Infrared Imaging System (Li-Cor). SDS-PAGE (10%).

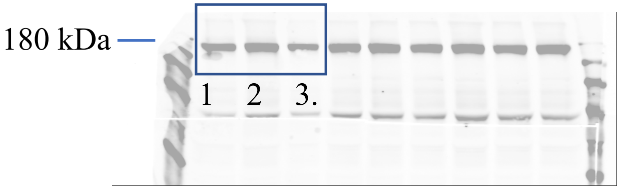

Fig. 4A. HepG2 whole cell lysate TFR. The membrane was cut into halves. The top part was blotted with a mouse anti-transferrin receptor (TFR) antibody (BD Biosciences). Antibody binding was detected using IRDye®680 or IRDye®800-labeled donkey anti-mouse, rabbit, or goat IgG (Li-Cor), followed by imaging on a Licor Odyssey Infrared Imaging System (Li-Cor). SDS-PAGE (8%).

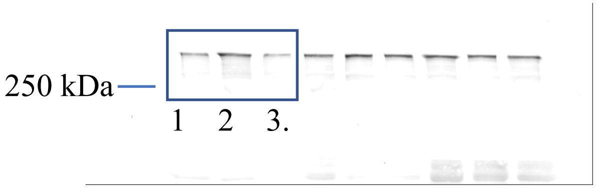

Fig. 4A. HepG2-apoB. The membrane was cut into halves. The top part was blotted with a goat anti-apoB antibody (Millipore Sigma). Antibody binding was detected using IRDye®680 or IRDye®800-labeled donkey anti-mouse, rabbit, or goat IgG (Li-Cor), followed by imaging on a Licor Odyssey Infrared Imaging System (Li-Cor). SDS-PAGE (6%).

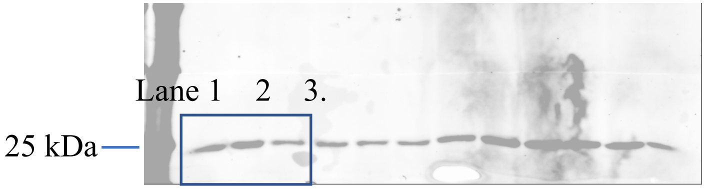

Fig. 4A. HepG2-apoA-I. The membrane was cut into halves. The bottom part was blotted with a mouse anti-apoA-I antibody (Cell signaling). Antibody binding was detected using IRDye®680 or IRDye®800-labeled donkey anti-mouse, rabbit, or goat IgG (Li-Cor), followed by imaging on a Licor Odyssey Infrared Imaging System (Li-Cor). SDS-PAGE (12%).

Figure 4 A-Medium-HepG2

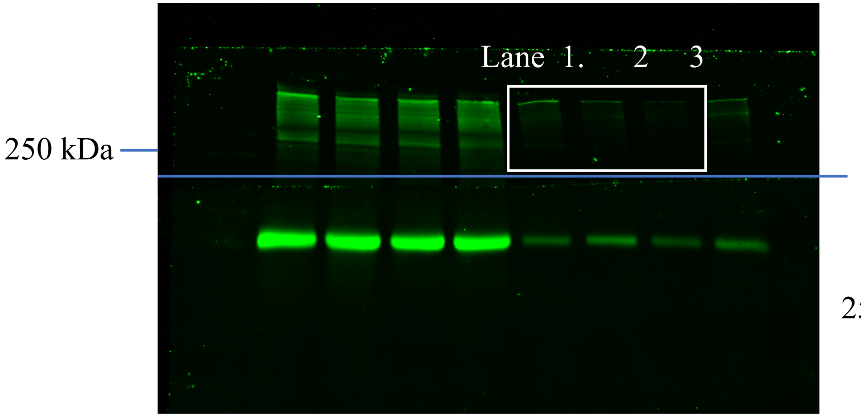

Fig 4A. HepG2 medium apoB. The membrane was cut into halves. The top part was blotted with a goat-anti apoB antibody (Millipore Sigma). Antibody binding was detected using IRDye®800-labeled donkey anti-goat IgG (Li-Cor), followed by imaging on a Licor Odyssey Infrared Imaging System (Li-Cor). SDS-PAGE (6%).

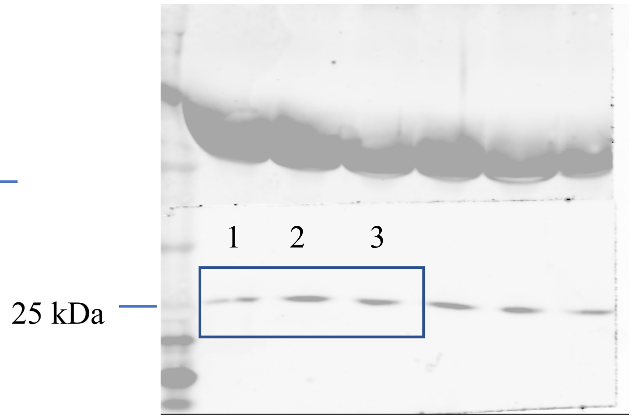

Fig. 4A. HepG2-medium apoA-I. The membrane was cut into halves. The bottom part was blotted with a mouse anti apoA-I antibody (Cell signaling). Antibody binding was detected using IRDye®680-labeled donkey anti-mouse IgG (Li-Cor), followed by imaging on a Licor Odyssey Infrared Imaging System (Li-Cor). SDS-PAGE (12%).

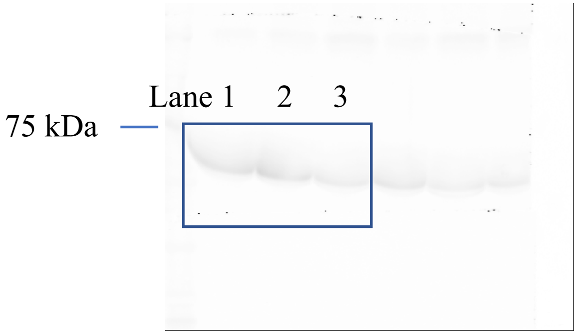

Fig. 4A. HepG2 medium albumin. The membrane was cut into halves. The top part was blotted with a rabbit anti-albumin antibody (abcam). Antibody binding was detected using IRDye®680-labeled donkey anti-rabbit IgG (Li-Cor), followed by imaging on a Licor Odyssey Infrared Imaging System (Li-Cor). SDS-PAGE (12%).

Figure 4 D-Huh7

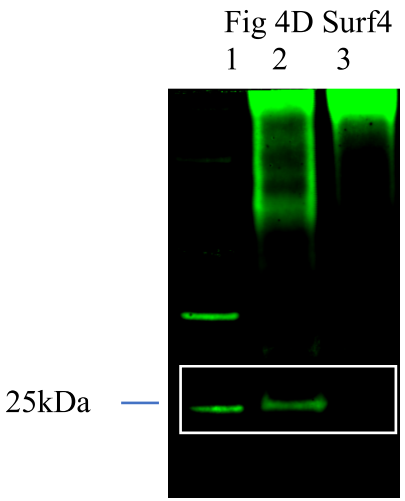

Fig 4D. Surf4. IP samples from Huh7 were subjected to SDS-PAGE (12%).The membrane was blotted with an anti-Surf4 antibody (Green). Antibody binding was detected using IRDye®800-labeled donkey anti-rabbit (Li-Cor), followed by imaging on a Licor Odyssey Infrared Imaging System (Li-Cor).

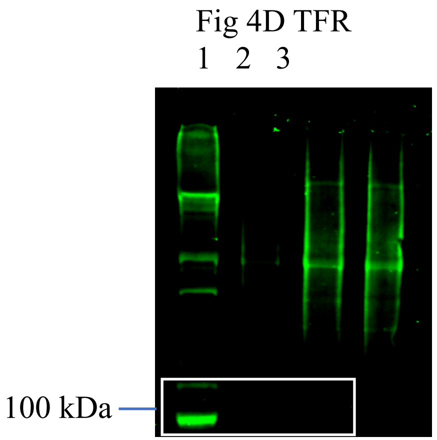

Fig. 4D. Transferrin receptor (TFR). IP samples from Huh7 cells were subjected to SDS-PAGE (4-12 %).The membrane was cut into halves. The top part was blotted with an anti-transferrin receptor antibody (TFR, BD Biosciences). Antibody binding was detected using IRDye®800-labeled donkey anti-mouse IgG (Li-Cor), followed by imaging on a Licor Odyssey Infrared Imaging System (Li-Cor).

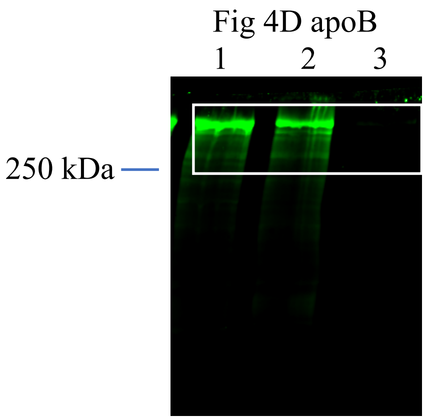

Fig 4D. apoB. IP samples from Huh7 cells were subjected to SDS-PAGE (6%).The membrane was cut into halves. The top part was blotted with a goat anti-apoB antibody (Millipore Sigma). Antibody binding was detected using IRDye®680 labeled donkey anti-goat IgG (Li-Cor), followed by imaging on a Licor Odyssey Infrared Imaging System (Li-Cor).

Figure 4 D-HepG2

Fig. 4D Surf4

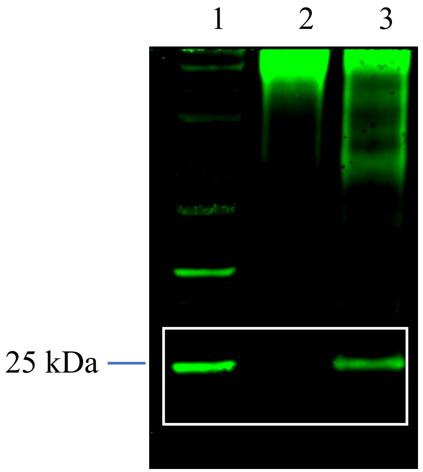

Fig. 4D. Surf4. IP samples from HepG27 were subjected to SDS-PAGE (12%).The membrane was blotted with an anti-Surf4 antibody (Green). Antibody binding was detected using IRDye®800-labeled donkey anti-rabbit (Li-Cor), followed by imaging on a Licor Odyssey Infrared Imaging System (Li-Cor).

Fig. 4D apoB

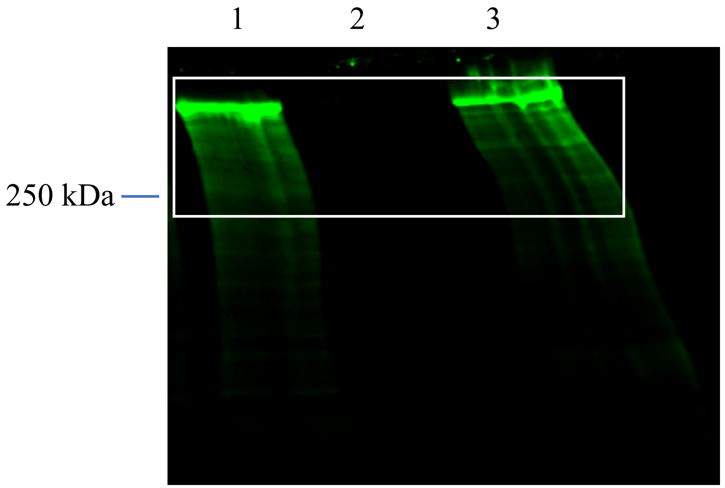

Fig. 4D. apoB. IP samples from HepG2 were subjected to SDS-PAGE (6%).The membrane was cut into halves. The top part was blotted with a goat anti-apoB antibody (Millipore Sigma). Antibody binding was detected using IRDye®680 labeled donkey anti-goat IgG (Li-Cor), followed by imaging on a Licor Odyssey Infrared Imaging System (Li-Cor).

Fig. 4D.TFR

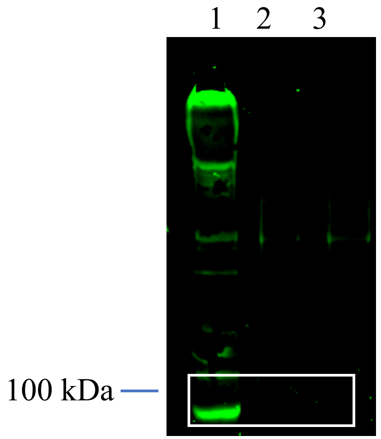

Fig. 4D. Transferrin receptor (TFR). IP samples from HepG2 were subjected to SDS-PAGE (4-12%).The membrane was cut into halves. The top part was blotted with an anti-transferrin receptor antibody (TFR, BD Biosciences). Antibody binding was detected using IRDye®800-labeled donkey anti-mouse IgG (Li-Cor), followed by imaging on a Licor Odyssey Infrared Imaging System (Li-Cor).

Figure 6F

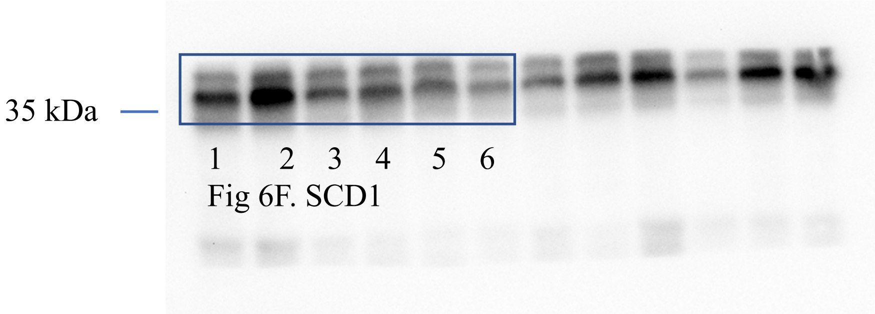

Fig 6F. SCD1. The membrane was cut into halves. The bottom part was blotted with a mouse anti-SCD1 antibody (abcam). Antibody binding was detected using HRP-conjugated secondary antibodies. SDS-PAGE (10%).

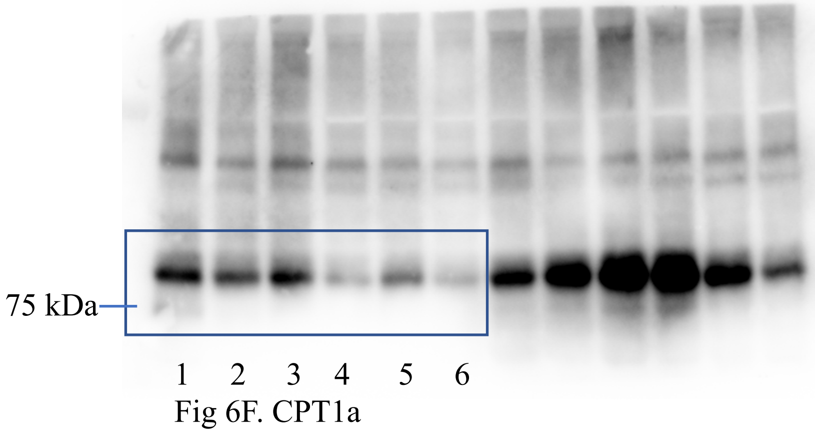

Fig 6F. CPT1a. The membrane was cut into halves. The bottom part was blotted with a mouse anti-CPT1a antibody (abcam). Antibody binding was detected using HRP-conjugated secondary antibodies. SDS-PAGE (10%).

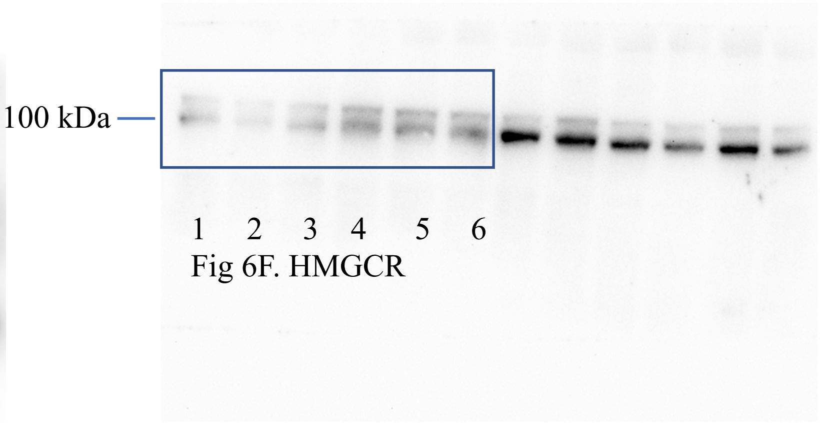

Fig 6F. HMGCR. The membrane was blotted with a rabbit anti-HMGCR antibody (abcam). Antibody binding was detected using HRP-conjugated secondary antibodies. SDS-PAGE (10%).

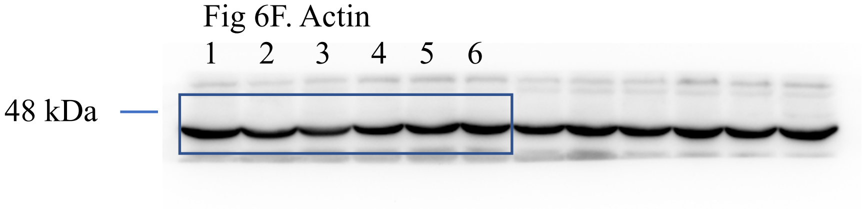

Fig 6F. Actin. The membrane was cut into halves. The bottom part was blotted with a rabbit anti-actin antibody (Bioss). Antibody binding was detected using HRP-conjugated secondary antibodies. SDS-PAGE (10 %).

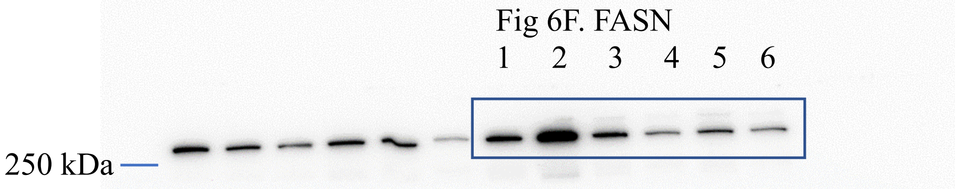

Fig 6F. FASN. The membrane was blotted with an anti-FASN antibody. Antibody binding was detected using HRP-conjugated secondary antibodies. SDS-PAGE (8%).

Figure 7C

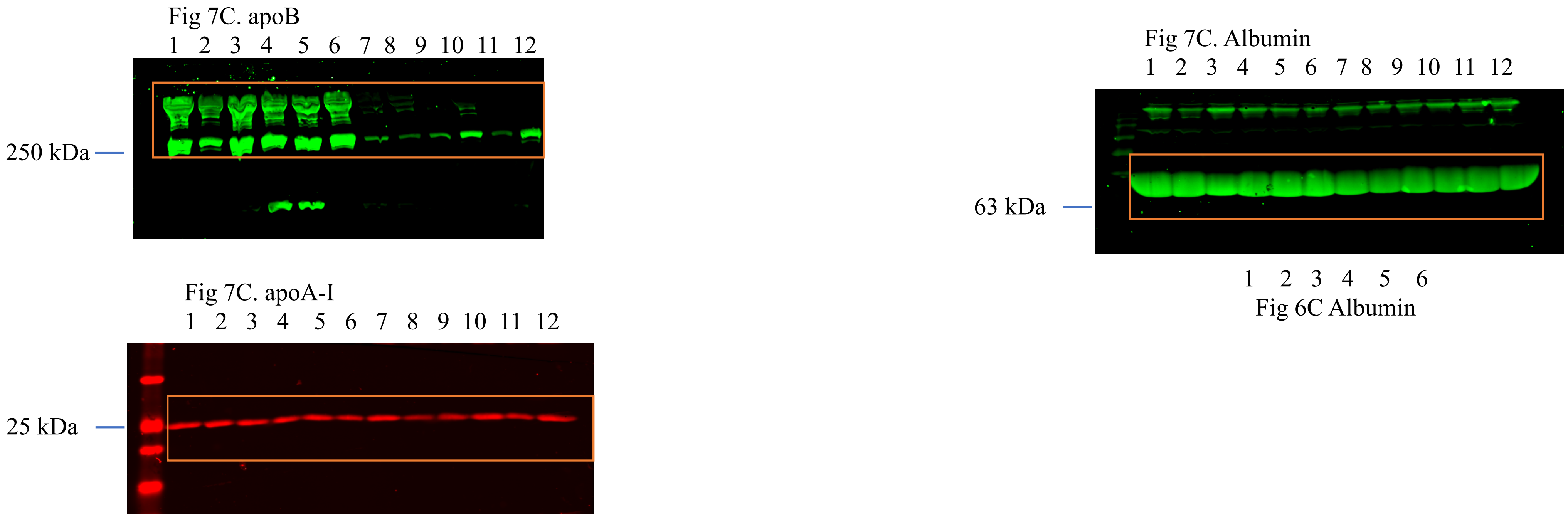

Fig 7C. apoB
1 2 3 4 5 6 7 8 9 10 11 12
250 kDa
Fig 7C. apoA-I
1 2 3 4 5 6 7 8 9 10 11 12
25 kDa
Fig 7C. Albumin
1 2 3 4 5 6 7 8 9 10 11 12
63 kDa
1 2 3 4 5 6
Fig 6C Albumin

Figure 7D

Fig. 7D apoB

1 2 3 4 5 6

250 kDa

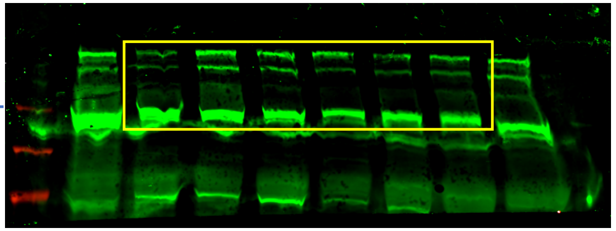

Fig. 7D apoA-I

1 2 3 4 5 6

25 kDa

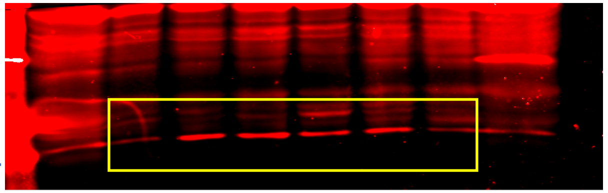

Fig. 7DAlbumin

1 2 3 4 5 6

63 kDa

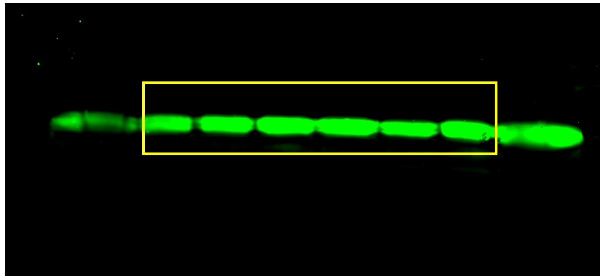

Fig. 7D Calnexin

1 2 3 4 5 6

75 kDa

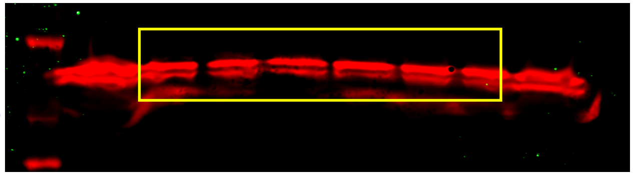

Figure 8F

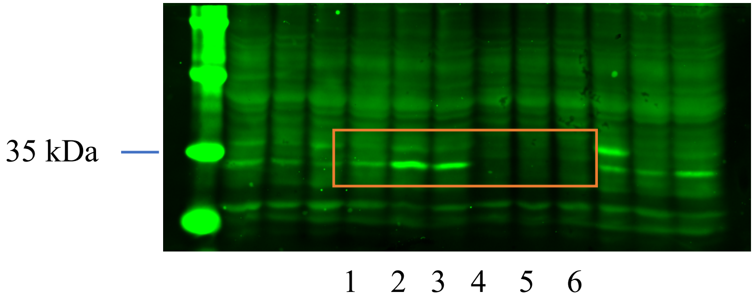

Fig. 8F SCD1

Fig. 8F Calnexin 1 2 3 4 5 6

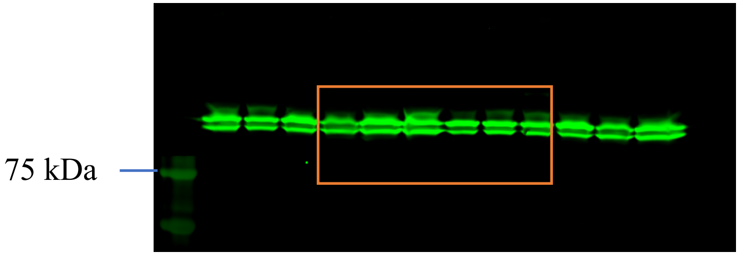

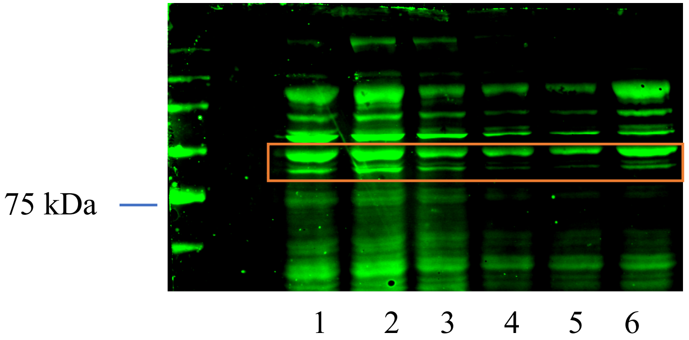

Fig. 8F CPT1a

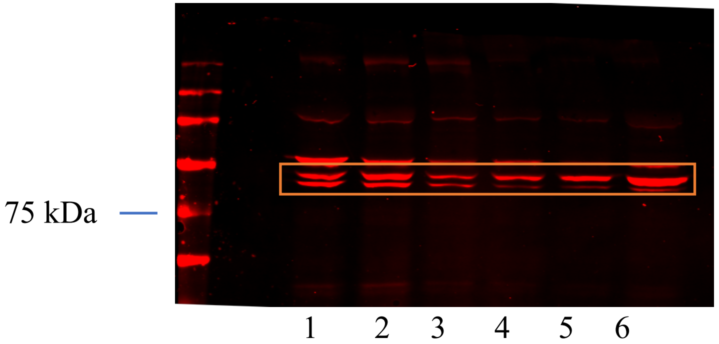

Fig. 8F HMGCR
